# 17q21.31 sub-haplotypes underlying H1-associated risk for Parkinson’s disease are associated with *LRRC37A/2* expression in astrocytes

**DOI:** 10.1186/s13024-022-00551-x

**Published:** 2022-07-15

**Authors:** Kathryn R. Bowles, Derian A. Pugh, Yiyuan Liu, Tulsi Patel, Alan E. Renton, Sara Bandres-Ciga, Ziv Gan-Or, Peter Heutink, Ari Siitonen, Sarah Bertelsen, Jonathan D. Cherry, Celeste M. Karch, Steven J. Frucht, Brian H. Kopell, Inga Peter, Y. J. Park, International Parkinson’s Disease Genomics Consortium (IPDGC), Alexander Charney, Towfique Raj, John F. Crary, A. M. Goate

**Affiliations:** 1 Department of Genetics and Genomic Sciences, Icahn School of Medicine at Mount Sinai, New York, NY USA; 2 Ronald M. Loeb Center for Alzheimer’s Disease, Icahn School of Medicine at Mount Sinai, New York, NY USA; 3 Laboratory of Neurogenetics, National Institute On Aging, National Institutes of Health, Bethesda, MD USA; 4 Department of Human Genetics, McGill University, Montréal, Québec Canada; 5 The Neuro (Montreal Neurological Institute-Hospital), McGill University, Montréal, Québec Canada; 6 Department of Neurology and Neurosurgery, McGill University, Montréal, Québec Canada; 7 Department for Neurodegenerative Diseases, Hertie Institute for Clinical Brain Research, University of Tübingen, Tübingen, Germany; 8 German Center for Neurodegenerative Diseases (DZNE), Tübingen, Germany; 9 Institute of Clinical Medicine, Department of Neurology, University of Oulu, Oulu, Finland; 10 Department of Neurology and Medical Research Center, Oulu University Hospital, Oulu, Finland; 11 Alzheimer’s Disease and CTE Center, Boston University, Boston University School of Medicine, Boston, MA USA; 12 Department of Neurology, Boston University School of Medicine, Boston, MA USA; 13 VA Boston Healthcare System, 150 S. Huntington Avenue, Boston, MA USA; 14 Department of Pathology and Laboratory Medicine, Boston University School of Medicine, Boston, MA USA; 15 Department of Psychiatry, Washington University in St Louis, St. Louis, MO USA; 16 Department of Neurology, Fresco Institute for Parkinson’s and Movement Disorders, New York University Langone, New York, NY USA; 17 Department of Neurosurgery, Icahn School of Medicine at Mount Sinai, New York, NY USA; 18 Center for Neuromodulation, Icahn School of Medicine at Mount Sinai, New York, NY USA; 19 Icahn Genomics Institute, Icahn School of Medicine at Mount Sinai, New York, NY USA; 20 Institute for Exposomic Research, Icahn School of Medicine at Mount Sinai, New York, NY USA; 21 Department of Psychiatry, Icahn School of Medicine at Mount Sinai, New York, NY USA; 22 IPDGC Authors and Affiliations in Supplementary Material 2, New York, NY USA; 23 Estelle and Daniel Maggin Department of Neurology, Icahn School of Medicine at Mount Sinai, New York, NY USA; 24 Department of Pathology, Icahn School of Medicine at Mount Sinai, New York, NY USA

**Keywords:** 17q21.31, Parkinson’s disease, LRRC37A, Copy number variation, Astrocytes

## Abstract

**Background:**

Parkinson’s disease (PD) is genetically associated with the H1 haplotype of the *MAPT* 17q.21.31 locus, although the causal gene and variants underlying this association have not been identified.

**Methods:**

To better understand the genetic contribution of this region to PD and to identify novel mechanisms conferring risk for the disease, we fine-mapped the 17q21.31 locus by constructing discrete haplotype blocks from genetic data. We used digital PCR to assess copy number variation associated with PD-associated blocks, and used human brain postmortem RNA-seq data to identify candidate genes that were then further investigated using in vitro models and human brain tissue.

**Results:**

We identified three novel H1 sub-haplotype blocks across the 17q21.31 locus associated with PD risk. Protective sub-haplotypes were associated with increased *LRRC37A/2* copy number and expression in human brain tissue. We found that LRRC37A/2 is a membrane-associated protein that plays a role in cellular migration, chemotaxis and astroglial inflammation. In human substantia nigra, LRRC37A/2 was primarily expressed in astrocytes, interacted directly with soluble α-synuclein, and co-localized with Lewy bodies in PD brain tissue.

**Conclusion:**

These data indicate that a novel candidate gene, *LRRC37A/2*, contributes to the association between the 17q21.31 locus and PD via its interaction with α-synuclein and its effects on astrocytic function and inflammatory response*.* These data are the first to associate the genetic association at the 17q21.31 locus with PD pathology, and highlight the importance of variation at the 17q21.31 locus in the regulation of multiple genes other than *MAPT* and *KANSL1*, as well as its relevance to non-neuronal cell types.

**Supplementary Information:**

The online version contains supplementary material available at 10.1186/s13024-022-00551-x.

## Background

**Fig. 1:**
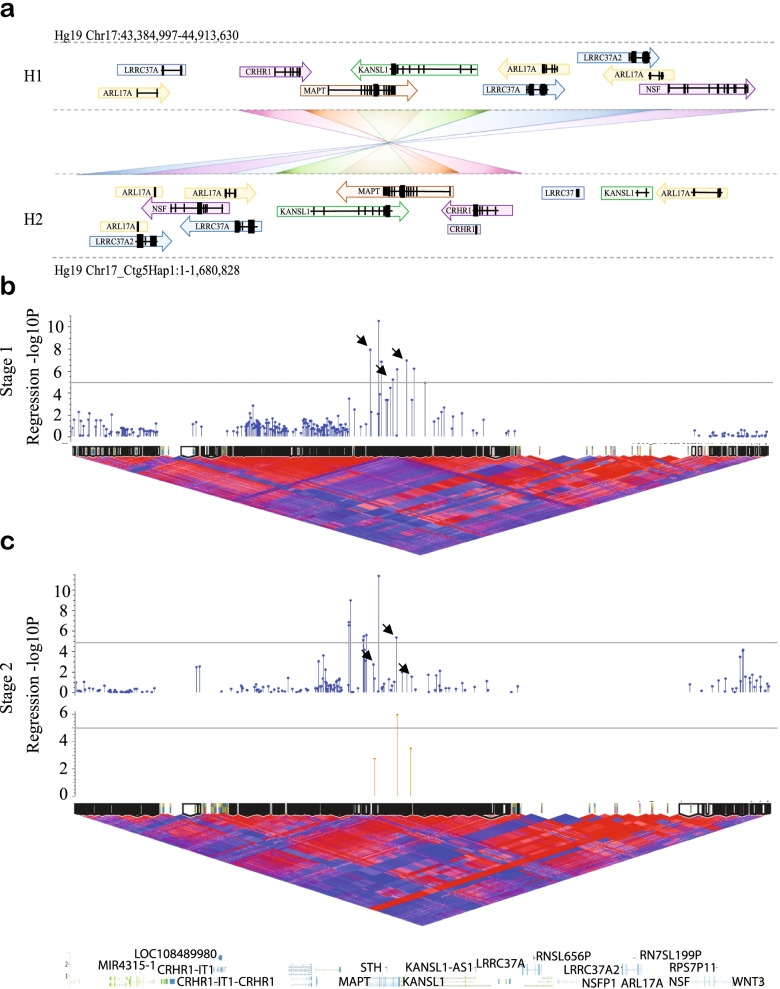
H1 sub-haplotypes within the *MAPT* 17q21.21 inversion region are associated with Parkinson’s disease risk. **A.** Structure of the 17q21.31 locus, which confers two distinct sub-haplotypes defined by gross structural inversion; H1 and H2. Direction of gene orientation in each haplotype is indicated by arrows. Each gene or partial gene is labeled with a distinct color and connected with a crossed rectangle between H1 and H2 to aid visualization of altered gene position between haplotypes. **B-C.** H1 sub-haplotype block association (-log_10_ *p*-value) with PD plotted above H1 homozygote D’ LD structure and sub-haplotype blocks generated from ***B***. Stage 1 data and ***C.*** Stage 2 data, spanning Hg19 Chr17:43,384,997–44,913,630. **C**. Association (-log_10_ *p*-value) of blocks calculated in Stage 2 data (blue, top), and H1.1, H1.2 and H1.3 blocks as defined in Stage 1 applied to Stage 2 data (orange, bottom). In LD plots, red indicates high D’ and blue indicates low. Black arrows indicate similar blocks generated across Stage 1 and Stage 2 data. Grey lines indicate genome wide suggestive significance *p*-value of 1 × 10^–5^

The *MAPT* 17q21.31 locus lies within a 1.5 Mb inversion region of high linkage disequilibrium (LD), conferring two distinct haplotypes; H1, which has a frequency of ~ 0.8 in European ancestry populations, and the less common, inverted H2 haplotype (frequency ~ 0.2), which is absent or lower frequency in East and South Asian populations (frequency 0—0.09) (Fig. 1A). The major haplotype, H1, has been genetically associated with increased risk for multiple neurodegenerative disorders, including *APOE* ɛ4-negative Alzheimer’s disease (AD) [1], corticobasal degeneration (CBD) [2], progressive supranuclear palsy (PSP) [3–5] and Parkinson’s disease (PD) [6–10].

PD is a movement disorder that commonly involves executive dysfunction and dementia [11, 12], but is classically characterized by bradykinesia, tremor, rigidity, postural instability, and numerous non-motor symptoms [13]. Neuropathologically, PD is an α-synucleinopathy defined by the presence of intraneuronal accumulation of α-synuclein in Lewy bodies throughout the substantia nigra, brainstem and forebrain [14, 15]. Despite the genetic association with the 17q21.31 *MAPT/*tau locus, aggregated tau is not a typical neuropathological feature of PD [15], although it is not rare for tauopathy to occur alongside α-synuclein inclusions in the substantia nigra [16, 17]. There is also no apparent association between PD and the H1c sub-haplotype [18, 19], which is strongly associated with risk for the primary tauopathies PSP and CBD [2, 3], indicating that different 17q21.31 locus variants and mechanisms may underlie the relative risk for each disease. Indeed, the 17q21.31 locus spans 1.5 Mb including multiple genes in addition to *MAPT*, and comprises several sub-haplotypes, defined by complex rearrangements and copy number variation in their distal regions [20, 21], the functional impact of which has not yet been explored. Indicative of this complexity, the 17q21.31 locus is genetically associated with many different phenotypes in addition to neurodegenerative diseases, the mechanisms for which remain largely unknown.

Given the specific loss of midbrain dopaminergic neurons in PD, this cell type has been the main focus of efforts to understand the mechanism of neuronal loss. Similarly, investigation of 17q21.31 variants is also often restricted to neurons, due to the high neuronal expression of *MAPT*. However, a causal role for astrocytes in PD pathogenesis has been recently proposed [22–25]. Indeed, many PD-associated genes are expressed in astrocytes and have known functional roles in astrocyte biology[24]. Furthermore, α-synuclein-positive aggregates have been identified in the cytoplasm of astrocytes in PD [26–28], indicating aberrant protein accumulation occurring as a result of either neuronal-glial transmission [22, 28, 29], or increased expression of endogenous α-synuclein in astrocytes themselves [25].

Although PD, like PSP and CBD, is associated with the H1 haplotype, the odds ratio for risk is substantially lower (1.5 vs 4.5) in PD [2–10]. Furthermore, in contrast to PSP and CBD, PD has not been associated with the H1c sub-haplotype [18, 19] and is not generally associated with the accumulation of tau. Together these observations suggest that the PD genetic association with the 17q21.31 locus may be driven by genes other than *MAPT*. Here, we report three sub-haplotype blocks associated with PD risk within the 17q21.31 H1 haplotype clade, with both protective and risk-associated sub-haplotypes within each block. We show that protective sub-haplotypes are associated with increased expression and copy number of *LRRC37A/2*, which we demonstrate is an astrocyte-enriched membrane-associated protein with a role in chemotaxis and the inflammatory response, and co-localizes with both soluble α-synuclein and Lewy bodies in human substantia nigra. These findings link the genetic association at the 17q21.31 locus with PD pathology, and support the hypothesis of astroglial dysfunction as a key contributing factor to PD disease pathogenesis.

## Results

### PD risk is associated with the 17q21.31 H1 haplotype

To confirm the genetic association of the 17q21.31 H1 haplotype with PD risk in our specific cohorts, we carried out a case–control association analysis across the region of interest (Figure S1A-E, Table S1) in two independent datasets (Stage 1 2,780 PD cases, 6,384 controls; Stage 2 2,699 cases, 2,230 controls; Table S2). The SNP with the strongest association in Stage 1 (rs17763050, *p* = 2.74 × 10^–9^) was in high LD with the known H1/H2 haplotype tag SNP rs8070723 (D’ = 0.98, Figure S1B-C, F). Both SNPs were associated with odds ratios (ORs) ~ 0.8 (95% CI ± 0.1; Figure S1B-C, Table S1), consistent with previously reported effect sizes^6,8^. Due to a smaller cohort size and consequent lack of power, the association with 17q21.31 was less prominent in Stage 2 data (Figure S1D-E, Table S1), and the top SNP did not tag the H2 haplotype. However, meta-analysis of both cohorts confirmed a significant association between PD risk and the 17q21.31 locus (Figure S1B-C, Table S1), with the H2 haplotype conferring protection (OR 0.82 (95% CI 0.76–0.89)) and the H1 haplotype therefore associated with increased risk.

As the major H1 haplotype was associated with increased risk for PD, we repeated the association analysis in H1 homozygotes alone in order to identify variants of H1 that may confer additional risk for PD (Figure S1G-J, Table S1). While association across the 17q21.31 locus was weaker in H1 homozygotes compared to the full data set, we observed a distinct signal spanning *MAPT* and *KANSL1* in Stage 1 and Stage 2 analyses (Figure S1G-H). The Stage 1 top SNP, rs41543512, was associated with an OR of 1.21 (95% CI 1.10–1.32, *p* < 0.001; Figure S1I, Table S1), but did not reach statistical significance in Stage 2 data or meta-analysis (Table S1). The most significant SNP in the Stage 2 analysis (rs139217062) was not present in Stage 1 data, although the second most significant variant from Stage 2, rs16940711, was not significant in Stage 1 data or by meta-analysis (Table S1, Figure S1J). These data suggest that the H1 association with PD may be more complex than variation in individual SNPs.

### 17q21.31 H1 sub-haplotype blocks spanning *MAPT* & *KANSL1* are associated with PD risk

As SNP-based association analyses were unsuccessful in identifying significant H1 sub-haplotypic variants contributing to PD risk, we decided to leverage the presence of high linkage disequilibrium (LD) within this region to investigate sub-haplotype blocks. We calculated discrete sub-haplotype blocks spanning the 17q21.31 locus using the D’ measure of LD, and performed a logistic regression association analysis on each block (Fig. 1B-C, Table 1). This approach greatly improved the power to detect disease-associated H1 variants in both Stage 1 and Stage 2 data. In Stage 1, we observed a peak spanning *MAPT* and the first 5 exons of *KANSL1* (Fig. 1B) that reached the genome-wide suggestive significance threshold of *p* = 1 × 10^–5^. Within this peak, three sub-haplotype blocks showed substantial overlap of SNPs in independently calculated blocks in Stage 2 data (Stage 1 blocks H1.1 (*p* = 1.73 × 10^–6^), H1.2 (*p* = 2.4 × 10^–4^) and H1.3 (*p* = 1.05 × 10^–5^); Fig. 1C, Table 1). We then applied the Stage 1-constructed blocks to Stage 2 data and observed replication of their association with PD risk (Fig. 1C, Table 1). Block H1.2 was highly significant in Stage 2 data (*p* = 1.12 × 10^–9^), while both blocks H1.1 and H1.3 were nominally significant (*p* < 0.002 and *p* < 0.0003, respectively).

**Table 1:** 17q21.31 H1 sub-haplotype blocks associated with PD susceptibility

							Stage 1			Stage 2		
Block	#Sub-haplotypes	#SNPs	Start	Stop	Gene	Location	*p*-value	-log_10_ *p*-value	FDR *p*-value	*p*-value	-log_10_ *p*-value	FDR *p*-value
H1.1	5	5	44,040,184	44,041,992	*MAPT*	Intronic	1.35E-08	7.87	1.73E-06	1.94E-03	2.71	4.50E-03
H1.2	8	8	44,090,196	44,097,249	*MAPT*	Intronic/Exonic	6.58E-06	5.18	2.40E-04	1.12E-09	5.95	7.84E-06
H1.3	7	9	44,119,987	44,131,305	*KANSL1*	Intronic	1.23E-07	6.91	1.05E-05	2.39E-04	3.47	1.12E-03

In order to determine whether the sub-haplotype blocks we identified were merely recapitulating previously defined *MAPT* sub-haplotypes [30], we estimated the frequency of each *MAPT* sub-haplotype in Stage 1 data using tag SNPs rs1467967, rs242557, rs3785883, rs2471738 and rs7521. We then overlaid the frequency of these *MAPT* sub-haplotypes with the frequency of each sub-haplotype across blocks H1.1, H1.2 and H1.3 (Figure S1K). We did not see any association between previously defined *MAPT* sub-haplotypes and block sub-haplotypes, although H1c was enriched in, but not exclusive to, protective block sub-haplotypes H1.1c, H1.2b and H1.3b (Figure S1K). Furthermore, there was no significant association between any *MAPT* sub-haplotype and PD risk in these data (Table S3, Figure S1L), indicating that the sub-haplotype blocks we have identified detect variation across the 17q21.31 locus associated with PD risk that are independent of previously defined *MAPT* sub-haplotypes.

Blocks H1.1, H1.2 and H1.3 each consist of multiple SNPs in high LD (Figure S1M) that generate multiple sub-haplotypes (Figure S2A-F). As many SNPs within these blocks were imputed, we confirmed the existence and frequency of each block and sub-haplotype using whole genome sequence (WGS) data from AMP-PD (Table S4, amp-pd.org), with the exception of H1.1d. This sub-haplotype occurred at a higher frequency than identified in Stage 1 data (~ 0.2 vs 0.06–0.1), and at an equivalent frequency to Stage 2 data (~ 0.2). Further investigation indicated this discrepancy was a result of skewed frequency of the H1.1d tag SNP rs16940758 in the American (NIH) cohort in Stage 1 data, but occurred at a similar frequency to the WGS data in the other three contributing datasets, suggesting a possible technical or imputation error for this variant. All other variants of interest and sub-haplotypes occurred at a similar frequency across cohorts and WGS data (Table S4), indicating accuracy of imputation and sub-haplotype construction.

Each sub-haplotype was differentially associated with PD susceptibility (Figure S2A,C,E, Table 2), with each block containing both risk- and protective sub-haplotypes with ORs ranging from 0.37 (95% CI 0.32–0.42, *p* = 1.3 × 10^–49^, H1.1c) to 2.51 (95% CI 2.2–2.86, *p* = 2.4 × 10^–45^, H1.1e). Despite the presence of heterogeneity between Stages 1 and 2, the most frequently occurring sub-haplotypes were replicated in both analysis stages and by fixed effects meta-analysis (Table 2). Specifically, we identified two sub-haplotypes in block H1.1; H1.1b and H1.1e, which increased risk in both datasets when compared against the most common haplotype, with ORs ranging from 1.26–1.6 (fixed effects *p* < 0.001) and 1.45–2.51 (fixed effects *p* < 0.001), respectively (Figure S2A-B, Table 2). In the same block we also observed a protective sub-haplotype (H1.1c), associated with an OR ranging 0.37–0.96 (fixed effects *p* < 0.001; Figure S2A-B, Table 2). Blocks H1.2 and H1.3 encompassed multiple sub-haplotypes with frequencies < 0.1, and exhibited greater variability and heterogeneity across stages. However, we identified one risk-associated sub-haplotype in block H1.2 (H1.2c, OR = 1.12–1.31, fixed effects *p* < 0.01) and two protective sub-haplotypes in block H1.3 (H1.3b; OR = 0.95–0.43, fixed effects *p* < 0.001, H1.3 g; OR = 0.55–0.83, fixed effects *p* < 0.002; Table 2, Figure S2C-F). These data reflect the variability and complexity present across the 17q21.31 locus, and indicate that numerous H1 variants exist that are associated with differential levels of risk for PD.

**Table 2:** H1 sub-haplotypes associated with PD susceptibility

			**Stage 1**			**Stage 2**			**Meta**					
**Block**	**Sub-haplotype ID**	**Sub-haplotype**	**Frequency (Case/Control)**	**OR** **(95% CI)**	**OR Fisher's exact *p***^**sig**^	**Frequency (Case/Control)**	**OR** **(95% CI)**	**OR Fisher's exact *p***^**sig**^	**RE OR (95% CI)**	**RE Heterogeneity (*p*)**	**RE p**^**sig**^	**FE OR (95% CI)**	**FE Heterogeneity (*p*)**	**FE p**^**sig**^
H1.1	H1.1a	ACTCT	0.25 (0.26/0.24)	1	-	0.21 (0.2/0.23)	1	-	1	-	-	1	-	-
	H1.1b	ACTTG	0.27 (0.38/0.22)	1.6 (1.45–1.78)	2.04E-19***	0.38 (0.39/0.36)	1.26 (1.09–1.46)	2.0E-03**	1.43 (1.13–1.81)	7.06 (0.0079)	3.0E-03**	1.48 (1.36–1.61)	7.06 (0.0079)	0***
	H1.1c	GCCTG	0.18 (0.09/0.23)	0.37 (0.32–0.42)	1.3E-49***	0.14 (0.13/0.16)	0.96 (0.79–1.15)	6.40E-01	0.59 (0.23–1.52)	67.79 (< 0.0001)	2.70E-01	0.51 (0.46–0.57)	67.85 (< 0.0001)	0***
	H1.1d	ATCTG	0.18 (0.06/0.24)	0.22 (0.19–0.26)	6.5E-92***	0.23 (0.24/0.21)	1.29 (1.1–1.52)	2.0E-03**	0.54 (0.1–3.01)	231.51 (< 0.0001)	4.90E-01	0.49 (0.44–0.55)	232.74 (< 0.0001)	0***
	H1.1e	ACCTG	0.12 (0.21/0.08)	2.51 (2.2–2.86)	2.4E-45***	0.02 (0.02/0.02)	1.45 (0.98–2.17)	5.70E-02	1.97 (1.15–3.36)	7.13 (0.0079)	1.3E-02*	2.37 (2.1–2.68)	7.13 (0.0076)	0***
H1.2	H1.2a	TTTCGATG	0.48 (0.44/0.5)	1	-	0.49 (0.49/0.48)	1	-	1	-	-	1	-	-
	H1.2b	TCTCGATG	0.17 (0.18/0.16)	1.27 (1.14–1.4)	2.02E-05***	0.17 (0.17/0.18)	0.93 (0.8–1.09)	3.56E-01	1.09 (0.81–1.47)	10.57 (0.001)	5.70E-01	1.14 (1.04–1.24)	10.58 (0.0011)	3.0E-03**
	H1.2c	TTAAAATA	0.15 (0.16/0.14)	1.31 (1.16–1.47)	4.52E-06***	0.19 (0.21/0.18)	1.12 (0.97–1.3)	1.21E-01	1.22 (1.05–1.42)	2.65 (0.103)	9.0E-03**	1.23 (1.13–1.35)	2.65 (0.1037)	4.41E-06***
	H1.2d	TTAAGATG	0.07 (0.08/0.06)	1.36 (1.16–1.59)	1.2E-04***	0.04 (0.03/0.05)	0.52 (0.38–0.69)	3.46E-06***	0.84 (0.33–2.19)	35.25 (< 0.0001)	7.27E-01	1.07 (0.94–1.23)	35.28 (< 0.0001)	2.90E-01
	H1.2e	CTTCGATG	0.06 (0.06/0.06)	1.17 (0.99–1.39)	5.64E-02	0.08 (0.08/0.08)	1.05 (0.85–1.3)	6.76E-01	1.12 (0.99–1.28)	0.72 (0.40)	7.00E-02	1.12 (0.99–1.28)	0.72 (0.397)	7.00E-02
	H1.2f	TTTCGGTG	0.05 (0.05/0.05)	1.16 (0.96–1.39)	1.18E-01	0.02 (0.02/0.03)	0.58 (0.41–0.83)	7.1E-03**	0.83 (0.42–1.64)	12.41 (0.0004)	6.00E-01	0.99 (0.84–1.16)	12.41 (0.0004)	9.10E-01
	H1.2 g	TTAAAATG	0.01 (0.01/0.01)	1.1 (0.77–1.54)	6.07E-01	-	-	-	-	-	-	-	-	-
	H1.2 h	TTTCGACG	0.01 (0.01/0.01)	1.28 (0.89–1.84)	1.64E-01	-	-	-	-	-	-	-	-	-
H1.3	H1.3a	GACTGAGAT	0.28 (0.32/0.27)	1	-	0.32 (0.32/0.32)	1	-	1	-	-	1	-	-
	H1.3b	CATTAGGGC	0.18 (0.11/0.22)	0.43 (0.38–0.49)	1.66E-41***	0.18 (0.18/0.19)	0.95 (0.81–1.11)	5.22E-01	0.64 (0.29–1.44)	58.53 (< 0.0001)	2.80E-01	0.57 (0.52–0.63)	58.57 (< 0.0001)	0***
	H1.3c	GATTGAGAT	0.17 (0.21/0.16)	1.11 (0.99–1.24)	7.37E-02	0.16 (0.16/0.17)	0.95 (0.80–1.12)	5.33E-01	1.04 (0.89–1.21)	2.24 (0.134)	6.10E-01	1.06 (0.96–1.16)	2.24 (0.1343)	0.23
	H1.3d	CTTTGGTGC	0.15 (0.12/0.16)	0.61 (0.54–0.7)	1.58E-14***	0.2 (0.21/0.18)	1.16 (0.99–1.36)	5.81E-02	0.84 (0.45–1.56)	35.27 (< 0.0001)	5.80E-01	0.77 (0.7–0.85)	35.28 (< 0.0001)	2.96E-07***
	H1.3e	CATTGGGGC	0.09 (0.14/0.07)	1.67 (1.45–1.92)	2.79E-13***	0.02 (0.02/0.02)	0.81 (0.54–1.21)	2.83E-01	1.19 (0.58–2.42)	12.01 (0.0004)	6.30E-01	1.54 (1.35–1.75)	12.01 (0.0005)	4.57E-11***
	H1.3f	CATTGGGGT	0.08 (0.07/0.09)	0.6 (0.51–0.70)	9.34E-11***	0.09 (0.1/0.08)	1.15 (0.93–1.42)	2.00E-01	0.81 (0.44–1.5)	21.37 (< 0.0001)	5.10E-01	0.74 (0.66–0.84)	21.38 (< 0.0001)	3.59E-06***
	H1.3 g	GATCGAGAT	0.04 (0.04/0.04)	0.83 (0.67–1.03)	9.39E-02	0.03 (0.02/0.02)	0.55 (0.38–0.78)	5.58E-04***	0.7 (0.49–1.02)	3.48 (0.062)	6.60E-02	0.75 (0.63–0.9)	3.48 (0.062)	1.6E-03**

*OR* Odds ratio, *CI* Confidence interval, *RE* Random effects meta-analysis, *FE* Fixed effects meta-analysis. *p < 0.05, **p < 0.01, ***p < 0.001

### PD-associated sub-haplotypes are associated with *LRRC37A/2* gene expression in human brain

**Fig. 2:**
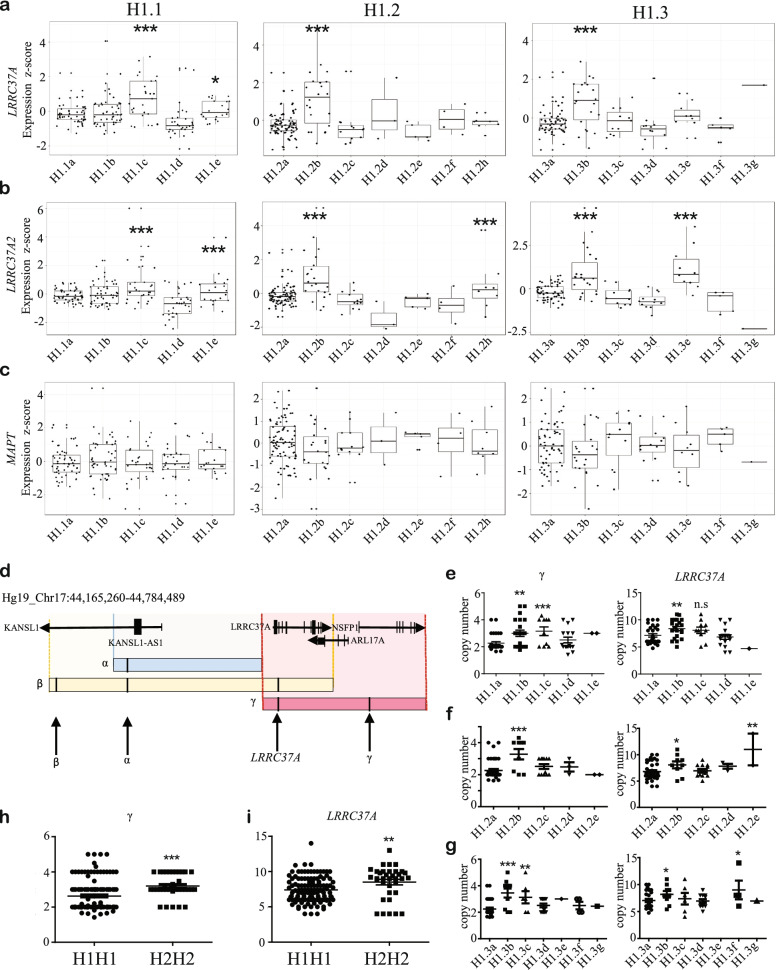
PD-associated sub-haplotypes are associated with *LRRC37A/2* expression and copy number **A-C**. Expression of ***A***. *LRRC37A, ****B.**** LRRC37A2* and ***C.**** MAPT* in human brain tissue, measured by RNA-seq across three different cohorts, split by sub-haplotype in blocks H1.1, H1.2 and H1.3. **D.** Schematic of the regions of copy number variation in the 3’ distal end of the 17q21.31 locus, as defined by Boettger et al. 2012[20]. Black arrows indicate the location of dPCR probes for (left to right) beta, alpha, *LRRC37A* and gamma. **E-G**. Copy number of gamma and *LRRC37A/2* regions in blocks ***E.*** H1.1, ***F.*** H1.2 and ***G.*** H1.3. **H-I**. Copy number of ***H.*** gamma and ***I.**** LRRC37A/2* regions between H1 and H2 homozygotes. All statistical comparisons are against the most common sub-haplotype. ns = not significant, *p < 0.05, **p < 0.01, ***p < 0.001.

In order to elucidate the functional consequences of PD-associated H1 block sub-haplotypes we queried publicly available post-mortem human brain RNA-seq data from dorsolateral prefrontal cortex (PFC) and temporal cortex (TCX) from the AMP-AD and CommonMind consortia (Table S5). Despite the position of the H1 association peaks across *MAPT* and *KANSL1*, we did not observe any differences in the expression of either of these genes between any sub-haplotypes in any block (Fig. 2C, Figure S3A-C). The only genes within the 17q21.31 locus that had a significant association with H1 PD-associated sub-haplotypes were *LRRC37A* and its paralog *LRRC37A2* (a.k.a *LRRC37A/2*; Fig. 2A-B, Figure S3D). We observed significantly increased *LRRC37A*/*2* expression in protective sub-haplotypes, specifically in H1.1c and H1.3b (H1.1c ~ 4.7 fold, *p* < 0.001, H1.3b ~ fivefold, *p* < 0.001), as well as in sub-haplotypes whose effects were not replicated across PD data-sets; H1.2b and H1.3e (H1.2b ~ 5, *p* < 0.001, H1.3e ~ 2.9x, *p* < 0.01), but were protective in the Stage 2 PD analysis (Fig. 2A-B). qRT-PCR on postmortem prefrontal cortex from a small number of individuals supported the observation of increased *LRRC37A/2* expression in these sub-haplotypes (Figure S3D).

We observed a significant reduction in *LRRC37A/2* expression in the PD risk-associated H1.1b sub-haplotype by qRT-PCR in human brain tissue (*p* < 0.01; Figure S3D), but not in the RNA-seq data. We also did not observe any difference in *MAPT* exons 2, 3 or 10 percent spliced in (PSI) values between sub-haplotypes (Figure S4A). Interestingly, *LRRC37A/2* expression was significantly higher in the protective 17q21.31 H2 haplotype (Figure S4B), which is consistent with our observation of increased *LRRC37A/2* expression in protective H1 sub-haplotypes. These data suggest that both the 17q21.31 H1/H2 and H1 sub-haplotype genetic associations with PD risk may be due to variable *LRRC37A/2* expression.

### Protective sub-haplotypes are associated with increased *LRRC37A/2* copy number

The 17q21.31 locus is structurally complex and encompasses regions of copy number variation (CNV) at its distal ends [20, 21]. As sub-haplotype blocks within *MAPT* and *KANSL1* were associated with altered *LRRC37A/2* expression, we tested whether they also tagged structural variants [20] in individuals homozygous for sub-haplotypes of interest (Fig. 2D-H, Figure S5A-D). Using DNA derived from either blood or brain tissue (Table S5) we performed digital PCR (dPCR) for *MAPT*, alpha, beta and gamma regions (Fig. 2D) [20], as well as for *LRRC37A/2* specifically (Fig. 2E-G, Figure S5A-D).

The majority of the structural variation in alpha and beta regions were found in the most common sub-haplotype for each block (H1.1a, H1.2a and H1.3a), with each subsequent sub-haplotype carrying fewer copies of these regions (Figure S5A-C). However, gamma and *LRRC37A/2* CNVs varied by sub-haplotype within each block; those sub-haplotypes exhibiting increased *LRRC37A/2* expression were also associated with significantly increased gamma (H1.1c R^2^ = 0.23, H1.2b R^2^ = 0.25, H1.3b R^2^ = 0.34, ~ 3–5 copies compared to 2–3 copies in controls, *p* < 0.05) and/or *LRRC37A/2* copy number (H1.1c R^2^ = 0.06, H1.2b R^2^ = 0.08, H1.3b R^2^ = 0.09, ~ 8–11 copies compared to 5–10 copies in controls, *p* < 0.05; Fig. 2E-G). Interestingly, risk-associated sub-haplotype H1.1b was associated with increased gamma and *LRRC37A/2* copy number (*p* < 0.01; Fig. 2E) but not *LRRC37A/2* expression (Fig. 2A-B), indicating that additional factors likely contribute to *LRRC37A/2* expression and PD risk in this locus, such as chromatin looping or variants within *LRRC37A/2* itself. Consistent with the expression data, we also observed increased beta, gamma and *LRRC37A/2* copy number in H2 homozygotes compared to H1 (Figure S5D). These data suggest that structural variation at the distal end of the 17q21.31 locus may underlie increased expression of *LRRC37A/2* in protective H2 and H1 sub-haplotypes.

### LRRC37A is a membrane-associated protein implicated in cellular migration, chemotaxis and inflammation

As PD sub-haplotypes converge on the expression and/or copy number of *LRRC37A/2*, we explored the likely function of this gene. We carried out RNA-seq analysis in HEK293T cells overexpressing *LRRC37A/2* in order to mimic increased copy number and to elucidate a potential function for this gene. The number of significantly differentially expressed protein-coding genes (fold change ± 1.5, adjusted *p* < 0.05) in the context of *LRRC37A/2* overexpression was minimal (28 upregulated, 21 downregulated), suggesting that *LRRC37A/2* is unlikely to play a major regulatory role. In order to confirm that we were not observing spurious changes in gene expression due to gross overexpression in a cell culture model, we carried out a titration of *LRRC37A/2* overexpression in HEK293T cells. We observed dose-dependent changes in the expression of genes that were significantly up or downregulated in our RNA-seq data (Figure S6A-D), confirming that the expression of these genes is likely to be altered by *LRRC37A/2* expression.

**Fig. 3:**
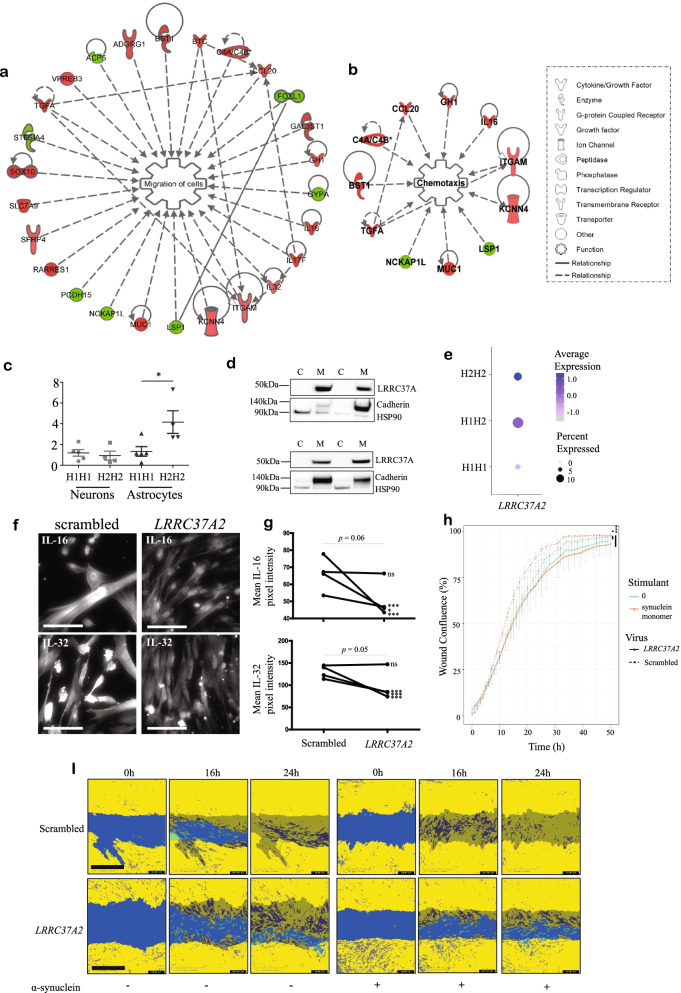
*LRRC37A/2* expression is associated with increased cellular migration, chemotaxis and inflammation. **A-B.** Significantly enriched ***A.**** Migration of cells* and ***B.**** Chemotaxis* pathways associated with *LRRC37A/2* overexpression, derived from Ingenuity pathway analysis. Red genes indicate upregulation, green indicates downregulation **C.** qRT-PCR for *LRRC37A* expression in H1 and H2 homozygote iPSC-derived neurons and astrocytes. *N* = 3 in duplicate. ns = not significant, *p < 0.05. **D.** Western blots for LRRC37A/2 in cytosolic (C) and membrane (M) fractions from (top) iPSC-neurons and (bottom) iPSC-astrocytes. Cytosolic fractions were confirmed by labeling with an anti-HSP90 antibody, and membrane fractions were confirmed by labeling with an anti-Pan-Cadherin antibody, *N* = 6. **E.** Proportion of *LRRC37A2*-expressing astrocytes by 17q21.31 haplotype from human prefrontal cortex snuc-seq data [33]. Dot size = proportion of cells expressing *LRRC37A2*, depth of color = *LRRC37A2* expression level. **F.** Representative immunofluorescence images of iPSC-derived astrocytes expressing either scrambled control or *LRRC37A2* shRNA lentivirus constructs, labeled with antibodies against either IL-16 or IL-32. Scale bar = 10 µm. **G.** Quantification of mean grey intensity of astrocytes in immunofluorescence images represented in ***F***. Each point represents the average of 12–16 individual images from an individual cell line. Four cell lines were analyzed. Points from the same iPSC line are connected between scrambled and *LRRC37A2* shRNA conditions. Overall *p*-value indicates paired t-test of cell line image means. Asterisks denote significant between scrambled and *LRRC37A2* conditions for each paired line. *p < 0.05, ***p < 0.001, ns = not significant. **H.** Wound confluence (%) of scratch wound repair over 50 h in scrambled control (dashed lines) and *LRRC37A2* shRNA (solid lines) treated iPSC-derived astrocytes treated with either 200 ng/ml α-synuclein monomers (orange) or PBS (green). Statistical difference between groups over time was determined by ANCOVA and post-hoc Tukey tests. *p < 0.05, ***p < 0.001. *N* = 4. **I.** Representative images with quantification masks of iPSC-astrocytes at 0 h,16 h and 24 h following induction of a scratch wound in scrambled control (top) and *LRRC37A2* shRNA (bottom) treated cells, with (right panels) and without (left panels) incubation with 200 ng/ml α-synuclein. Yellow masks indicate cell confluence, blue indicates the scratch wound area, and grey indicates cellular migration into the original wound area. Scale bar = 600 µm

Functional enrichment of gene ontology (GO) terms for significantly differentially expressed genes indicated a role for LRRC37A/2 at the cell membrane (Fig. 3A-B, Figure S6E-F), which we confirmed by western blot in HEK293T cells (Figure S6G). In addition, *LRRC37A/2* overexpression also resulted in significant enrichment for *cell communication* (GO:0,007,154, *p* < 0.05) and *neuroactive ligand-receptor interaction* (KEGG:04,080, *p* < 0.05) pathways, as well as nominal enrichment for membrane-component-related pathways (Figure S6F).

Ingenuity pathway analysis (IPA) also suggested a role for LRRC37A/2 at the plasma membrane and in the extracellular space; specifically, these analyses indicated that increased *LRRC37A/2* expression resulted in upregulated cellular movement pathways, such as increased *migration of cells* (*p* < 0.01, z score = 1.85; Fig. 3A) and upregulation of *chemotaxis* (*p* < 0.05, z score = 0.918; Fig. 3B). Several upregulated genes within these pathways are also essential in regulation of the inflammatory response; both *IL17F* and *IL32* are pro-inflammatory cytokines that mediate the inflammatory response of astrocytes [31, 32], whereas *IL16* acts as a chemoattractant for cells expressing CD4. These data therefore suggest that increased *LRRC37A/2* expression may mediate astroglial inflammation and modify cellular migration in response to a stimulant, such as α-synuclein.

### LRRC37A/2 is expressed in astrocytes and impacts cellular migration and response to α-synuclein

Our pathway analysis indicated that *LRRC37A/2* is associated with cellular signaling and pro-inflammatory pathways relevant to astroglial function. We therefore sought to confirm whether *LRRC37A/2* was expressed and functional in these cells. As *LRRC37A/2* expression and copy number was higher in 17q21.31 H2 haplotype carriers compared to H1, and due to difficulty in identifying multiple human iPSC lines with specific H1 sub-haplotypes, we compared the expression of *LRRC37A/2* and genes altered by *LRRC37A/2* expression in iPSC-derived neurons and astrocytes homozygous for either H1 or H2 haplotypes in order to identify the most relevant neural cell type (Fig. 3C, Figure S6H). While *LRRC37A/2* was expressed in both cell types, we observed increased expression of *LRRC37A/2* and associated genes in H2 astrocytes, but not in H2 neuronal cultures (Fig. 3C, Figure S6H), suggesting that H1/H2-associated *LRRC37A/2* expression changes may be specifically impacting astroglial gene expression and function. We also confirmed that LRRC37A/2 was present in the plasma membrane in both iPSC-derived neurons and astrocytes by isolating cytosolic and membrane-associated proteins from each cell type and analyzing the resulting fractions by western blot (Fig. 3D). We further confirmed that 17q21.31 haplotype may be more relevant to astrocyte function by comparing *LRRC37A/2* expression in single nuclei sequencing data (snuc-seq) from human prefrontal cortex [33] (Figure S6I). We found that *LRRC37A2* was more highly expressed in all cell types compared to *LRRC37A*, indicating that this may be the driver behind any functional differences due to haplotype (Figure S6J). Furthermore, there was a clear H2 dose-dependent effect on the proportion of *LRRC37A2*-expressing astrocytes and level of expression (Fig. 3E) although due to the small population of astrocytes in this dataset (Figure S6I), the difference in *LRRC37A2* expression between H1H1 and H2H2 astrocytes did not pass multiple test correction. In contrast, while there were fewer *LRRC37A2*-expressing cells in H1H1 cells compared to H1H2 and H2H2, there was no dose-dependent effect of the H2 haplotype on *LRRC37A2* expression in either excitatory or inhibitory neuronal populations (Figure S6K).

To further validate the function of *LRRC37A2* expression in astrocytes, we knocked down its expression in iPSC-derived astrocytes using lentiviral *LRRC37A2* shRNA, which was confirmed by qRTPCR (Figure S7A). We then confirmed that genes that were upregulated in HEK293T cells in response to *LRRC37A2* overexpression were reduced in astrocytes following *LRRC37A2* knockdown (Figure S7A), thus validating the relevance of our HEK293T gene expression data. In addition, consistent with the RNA-seq data, we then demonstrated significantly reduced expression of cytokines IL-32 and IL-16 in *LRRC37A2* knock-down astrocytes by immunofluorescence in 3/4 donor lines examined (Fig. 3F-G, Figure S7B-C). In order to determine whether *LRRC37A2* expression was involved in cellular migration and response to stimulants, we conducted a scratch wound repair assay in the presence or absence of monomeric α-synuclein and measured the rate of wound repair over the course of 50 h (Fig. 3H-I). In the absence of α-synuclein, there was no significant difference in the rate of wound repair (% wound confluence) over time between *LRRC37A2* shRNA and scrambled control groups (*p* > 0.05). Treatment with monomeric α-synuclein increased the rate of wound repair in scrambled control astrocytes (*p* = 0.05) indicating a responsiveness to α-synuclein as a stimulant. However, *LRRC37A2* knock down astrocytes demonstrated no such response to α-synuclein exposure, and their rate of wound repair was significantly slower than compared to controls treated with α-synuclein (*p* < 0.001) (Fig. 3H-I). These data confirm a likely role for *LRRC37A2* expression in disease-relevant astrocytic functions including inflammation, cellular migration and responsiveness to α-synuclein.

### LRRC37A2 interacts with soluble and aggregated α-synuclein in human substantia nigra.

**Fig. 4:**
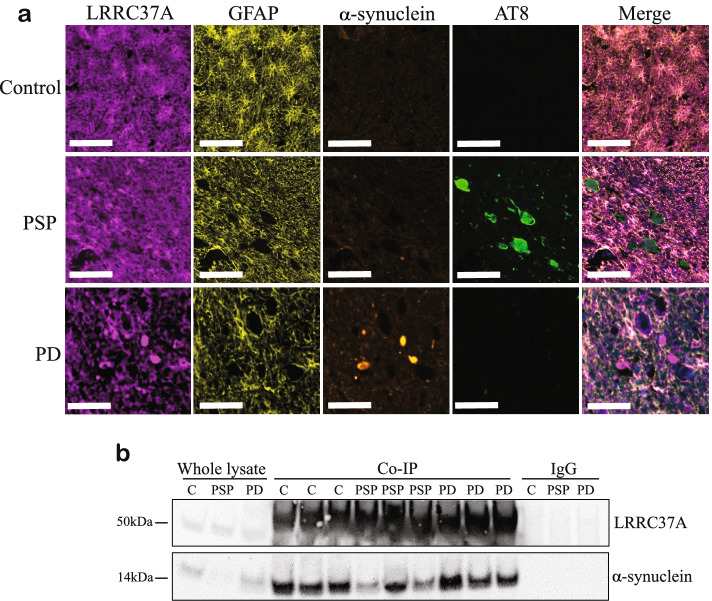
LRRC37A/2 is expressed in astrocytes in the substantia nigra and is co-localized with α-synuclein **A.** Representative images from multiplex immunofluorescent staining of human control, PSP and PD substantia nigra sections with astrocyte marker GFAP, LRRC37A, α-synuclein and pathologically phosphorylated Tau (AT8). Scale bar = 100 µm, *N* = 4-5. **B**. Co-immunoprecipitation (Co-IP) of LRRC37A (top panel) with soluble α-synuclein (bottom panel) in substantia nigra from control (C), PSP and PD brain, *N* = 3. Whole protein lysates and IgG only controls were included for comparison

In order to assess whether LRRC37A/2 was expressed in mature astrocytes in human brain tissue, we carried out multiplex immunofluorescence staining in human substantia nigra from PD, PSP and aged controls (Fig. 4A). We found that in all cases, LRRC37A/2 co-localized with the astrocyte marker GFAP, but not the microglia marker IBA1 (Fig. 4A, Figure S7D). In contrast, α-synuclein positivity was observed only in PD substantia nigra, and hyperphosphorylated tau (labeled with AT8) was present only in PSP brain (Fig. 4A). Interestingly, in regions with Lewy body pathology there was reduced staining intensity of LRRC37A/2 in astrocytes, and colocalization of LRRC37A/2 with Lewy bodies (Fig. 4A). However, there was no association between tau AT8 positivity and LRRC37A/2 expression in PSP substantia nigra (Fig. 4A), indicating that LRRC37A/2 accumulation is specific to PD pathology. To validate the association of LRRC37A/2 with α-synuclein, we performed co-immunoprecipitation from control, PSP and PD substantia nigra tissue (Fig. 4B). We found that soluble α-synuclein bound LRRC37A/2 in all cases, whereas IgG alone did not (Fig. 4B), indicating that LRRC37A/2 and α-synuclein likely form a complex in human brain that becomes disordered in the context of PD pathogenesis. Furthermore, analysis of publicly available snuc-seq data from PD and control midbrain [34] indicated a trend towards reduced *LRRC37A2* expression in PD astrocytes compared to controls (Figure S7E-F), consistent with a potentially protective effect of increased *LRRC37A/2* expression. These data are not only the first to identify a role for LRRC37A/2 in astrocytes, but are the first to link the genetic association at the 17q21.31 locus with PD pathology.

## Discussion

By constructing discrete sub-haplotype blocks across the 17q21.31 H1 locus, we have identified multiple novel H1 sub-haplotypes associated with variable levels of PD risk, independent of the previously constructed sub-haplotypes across *MAPT *[30]. The association of some of these blocks with PD risk was inconsistent between stage 1 and stage 2 analyses, possibly due to genotyping and imputation error. We were able to identify blocks and sub-haplotypes with common protective effects across populations. The *MAPT* gene, encoding the microtubule associated protein tau, is central to this locus and is likely the causal gene for other neurodegenerative disorders genetically associated with the H1 haplotype, such as PSP and CBD [2, 3], given that they are characterized neuropathologically by tau hyperphosphorylation and accumulation. Recently, reduced expression of *MAPT-AS1* has been identified in PD substantia nigra [38] and has been shown to have an inverse correlation with *MAPT* expression [39], suggesting a potential role for *MAPT* expression in PD pathogenesis. However, we did not observe any association between *MAPT* expression or splicing and 17q21.31 haplotype or sub-haplotypes in our analyses, indicating that any disease-associated *MAPT* regulation in PD brain may not be due to haplotype. Furthermore, while tau pathology can occur alongside α-synuclein inclusions in the substantia nigra [16, 17], it is not a typical neuropathological feature of PD [15]. The causal gene underlying the genetic association across the 17q21.31 locus with PD risk has therefore been unclear, although a recent study proposed that H1-associated variants in *KANSL1* alter mitophagy [40]. In contrast, we did not find any effect of our PD-associated H1 sub-haplotypes on either *KANSL1* expression or copy number, but did observe a consistent association with the expression of another gene in the locus, *LRRC37A/2*. However, these data do not rule out that *KANSL1* variants may alter disease risk independently of *LRRC37A/2* expression between the major haplotype clades and H1 sub-haplotypes.

We found that protective H1 sub-haplotypes were associated with increased expression of *LRRC37A/2*. This is consistent with increased expression of these genes in the protective H2 haplotype, suggesting that there is likely a shared mechanism of protection from PD between H2 and specific sub-haplotypes of H1. Furthermore, analysis of CNVs in the 3’ distal end of the 17q21.31 locus suggested that protective sub-haplotypes were tagging structural variants defined by increased gamma region [20] and *LRRC37A/2* copy number, which likely underlies the increased expression of these genes. *LRRC37A* is a core duplicon on chromosome 17 [41] and is present at the inversion breakpoint of the 17q21.31 locus; it has been hypothesized that its propensity for CNVs is responsible for the evolutionary toggling of this region that resulted in the distinct H1 and H2 haplotypes [42]. Due to the complex structural variation surrounding *LRRC37A* and the presence of its paralog *LRRC37A2*, it is challenging to genotype or sequence this region of the genome. As such, our analyses have not been able to separate the contribution of each gene and have considered them together, however *LRRC37A2* expression was consistently higher in both neurons and glial cells than *LRRC37A* and may therefore be the most relevant gene. As a consequence of the CNVs in this region, genotyping data across *LRRC37A* and *LRRC37A2* is of low confidence and quality, and as such is excluded from GWAS analyses, as is visible in any association plot, and likely explains why the peak genetic signal for PD association was close to *MAPT* and *KANSL1*. The association between *LRRC37A/2* variants and any disease or phenotype has therefore never been tested, and it is likely that additional variation within *LRRC37A/2* itself is contributing to its altered expression and function that may also impact PD risk. Little is known about the function of *LRRC37A/2*, although it has been associated with an increased immune and inflammatory response [43], as well as with cellular migration and synapse formation [41]. Despite conducting our RNA-seq analysis of *LRRC37A/2* overexpression in HEK293T cells, we observe enrichment of pathways consistent with these data; we found that increased *LRRC37A/2* expression upregulated cellular migration and chemotaxis pathways, which are both essential mechanisms involved in wound healing and inflammation. Furthermore, we validated these gene expression changes and demonstrated a role for *LRRC37A2* in astroglial migration in iPSC-derived astrocytes.

Within these pathways we observe increased expression of pro-inflammatory cytokines *IL17* and *IL32*, as well as the chemoattractant *IL16*, each of which are involved in the astrocytic inflammatory response [31, 32]. Neuroinflammation of the substantia nigra is considered a characteristic feature of PD in addition to neuronal loss [44, 45], and many genes associated with PD, such as *GBA*, *LRRK2* and *PINK1* are thought to have a role in the inflammatory response in astrocytes [24, 46]. Furthermore, the most significantly enriched pathway in our analysis, *Neuroactive-ligand receptor interaction*, is also involved in the inflammatory response and has previously been associated with PD; this pathway was significantly enriched in a functional assessment of PD GWAS signals [47], and is targeted by microRNAs that are upregulated in a *Drosophila* model of PD [48]. We also observe upregulation of *TGFA*, the infusion of which into the forebrain of a rat model of PD increased the proliferation of neuronal and glial progenitors and the production of dopaminergic neurons to the substantia nigra [49], indicating that this may be a protective growth factor against neuronal loss in PD. *LRRC37A/2* overexpression in HEK293T cells was therefore able to recapitulate pathways associated with PD, despite being a cell type with limited relevance to PD pathogenesis.

As these expression data were indicative of pathways relevant to astrocyte biology, and we found that *LRRC37A/2*-associated gene expression changes were apparent in astrocytes but not neurons, we hypothesized that this was likely the most relevant cell type for *LRRC37A/2* expression. Indeed, in human substantia nigra tissue we observe localization of LRRC37A/2 specifically in astrocytes. The contribution of astrocytic dysfunction to PD pathogenesis has gained attention in recent years, and is hypothesized to be a causal mechanism for the initiation and progression of PD [22–25]. Many genes associated with PD risk are expressed in astrocytes, the functions of which converge on the inflammatory response, lipid handling, mitochondrial health and lysosomal function [24]. Our finding that *LRRC37A/2* is expressed in astrocytes and plays a role in the inflammatory response is therefore consistent with known pathogenic mechanisms of PD. Furthermore, we demonstrate that *LRRC37A2* expression may play a role in the detection of extracellular monomeric α-synuclein by astrocytes, and consequently impacts migration and tissue repair. α-synuclein has previously been identified as a stimulant for astrocytes [50], inducing a reactive state [51]. This mechanism was impaired in the context of reduced *LRRC37A2* expression, indicating that appropriate detection and response to stimulants by astrocytes is likely a protective mechanism against neuronal death.

However, the role of astroglial inflammation in PD is unclear [22]. As a further complication, in vitro studies of human astrocyte cultures indicate that α-synuclein induces the release of pro-inflammatory cytokines [22, 25], but these cells also release protective molecules such as GDNF in response to dopaminergic neuronal damage, and such trophic support may benefit neuronal survival [22]. In addition, the substantia nigra is considered to be particularly susceptible in PD as dopaminergic neurons in this region are surrounded by the lowest proportion of astrocytes in the brain [52]. Whether an inflammatory response in this context would be protective or exacerbate neuronal death is therefore unknown. As our data suggest increased *LRRC37A/2* expression is protective and associated with increased expression of pro-inflammatory cytokines, astroglial inflammation in response to α-synuclein may therefore be protective.

Interestingly, we observe an interaction between LRRC37A/2 and soluble α-synuclein, as well as co-localization of LRRC37A/2 with Lewy bodies in PD substantia nigra. The function and mechanism of this interaction is untested, although it is likely that a complex is formed in astrocytes and propagated to neurons. iPSC-astrocytes have been reported as expressing low levels of endogenous α-synuclein, which is increased in cells derived from PD patients [25], and α-synuclein released from neuronal axon terminals is taken up by astrocytes [28], which can be further transferred to neurons [23, 29]. This raises the possibility that LRRC37A/2 may influence α-synuclein release, aggregation and/or propagation.

## Conclusions

In conclusion, we have identified novel sub-haplotype variants of the 17q21.31 H1 clade significantly associated with protection against PD. While the genetic association across this locus is typically ascribed to *MAPT* or *KANSL1*, we find evidence for the involvement of a novel gene, *LRRC37A/2,* in PD risk. We propose that in a similar mechanism to other PD-associated genes, *LRRC37A/2* is expressed in astrocytes and plays a role in the regulation of astroglial inflammation, specifically in the release of pro-inflammatory cytokines, chemotaxis and cellular migration. Importantly, we demonstrate that LRRC37A/2 interacts and co-localizes with α-synuclein and Lewy bodies, thus indicating a potential modifying role in the formation of PD pathology. These findings link the genetic association at the 17q21.31 H1 locus with PD pathology, and support the hypothesis of astroglial dysfunction as a key contributing factor to PD disease pathogenesis.

## Methods

### Genotype data treatment

Case and control data from several cohorts from the International Parkinson’s Disease Genetics Consortium (IPDGC; NIA, GER, FIN, NL, SP, McGill) [6, 9, 10] was kindly shared by Drs. Nalls, Singleton and Bandres-Ciga (NIH, Bethesda, MD; Tables S2, S6).

#### Pre-imputation QC

Each dataset was obtained with different QC filters already applied, and so were all subsequently passed through the same, more stringent QC pipeline to ensure consistency. Plink v1.9 [53] was used to perform quality control for all datasets. First, SNPs were filtered by a 98% call rate, and remaining SNPs with a MAF < 1% were excluded. Individuals with < 98% genotyping rate were then removed. To determine and correct for population stratification, principal components analysis was carried out in combination with Hapmap YRI, CEU and CHB populations [54] using EIGENSOFT [55]. Samples that did not cluster with the CEU European ancestry population were excluded. Identity by descent analysis was then conducted, and related individuals or potential sample duplicates (Z0 ≤ 0.8) were removed. We were unable to assess discordant sex information on data acquired from other sources, as the required information for this analysis was not provided to us. Variants that deviated from Hardy Weinberg equilibrium at a significance threshold < 1 × 10^–4^ were then removed. Chromosome 17 was then isolated and screened for strand mismatches.

### Imputation and post-imputation QC

Filtered chromosome 17 data from each cohort was submitted individually to the Michigan imputation server [56] (https://imputationserver.sph.umich.edu) and imputed against the HRC r1.1 2016 panel using Eagle v2.3 phasing. Following imputation, SNPs with an r2 < 0.3 were removed, and remaining SNPs were filtered for a 99% call rate. Genotyping call rates for individuals were again filtered at 99%, and SNPs that deviated from Hardy–Weinberg equilibrium at a significance threshold < 1 × 10^–6^ were excluded. Individual cohorts were then merged, and finally filtered once more with a SNP call rate of 99%. Prior to analysis, variants were filtered to exclude SNPs with a MAF < 0.01.

### Single SNP association analyses

Logistic regression association analyses using an additive model were carried out in SNP and Variation Suite v8.8.1 (SVS8) software (Golden Helix, Inc., Bozeman, MT, www.goldenhelix.com). As all potential covariate information was not available, the model was corrected using the first 10 principal components as calculated by SVS8. Associations were initially carried out on the entire cohort in order to confirm the 17q21.31 H1/H2 haplotype association. The data was then filtered for H1 homozygotes only, using tag SNP rs8070723 and the association analysis was repeated with the same parameters.

Meta-analysis of SNP effects across multiple datasets was carried out using the R package rmeta [57] using both Random Effects (DerSimonian-Laird) and Fixed Effects (Mantel–Haenszel) approaches. Calculation and visualization of linkage disequilibrium (LD) over large genomic ranges was carried out in SVS8 using both r2 and D’. Inspection of LD between individual SNPs of interest was carried out using Haploview [58].

### Haplotype block construction and association

Haplotype blocks were constructed in SVS8 using the D’ measure of LD. Blocks were defined using guidelines as described by Gabriel et al. (2002) [59]. Each block contained a maximum of 15 markers within 160 kb of each other, with a D’ upper confidence bound ≥ 0.98 and a lower confidence bound ≥ 0.7. Haplotypes were estimated using an expectation–maximization (EM) algorithm with 50 iterations, and a convergence tolerance of 0.0001. Sub-haplotypes with a frequency < 0.01 were excluded from further analysis. Case–control association analyses were carried out per block using a logistic regression model. Odds ratios and associated Fisher’s exact p-values were calculated for each sub-haplotype within each block using the R package epitools [60].

*MAPT* sub-haplotypes were defined as previously described [30, 61]. Sub-haplotypes were estimated for each individual in Stage 1 data as described above, and case–control association analysis was conducted for association with each sub-haplotype as described above.

### WGS data

A subset of processed whole genome sequencing data was obtained from the AMP-PD Parkinson’s Progression Markers Initiative (PPMI) (amp-pd.org/unified-cohorts/ppmi). Data were further processed using a publicly available, in-house WGS QC pipeline (https://github.com/ricardovialle/WGS-QC-Pipeline). Briefly, data were filtered to exclude variants with MAF < 1%, missingness > 5%, read depth < 200,000, mapping quality < 58.75 and > 61.25, and with an inbreeding coefficient < -0.8. Samples were excluded with a missingness > 10% and relatedness threshold > 0.125. This resulted in a final dataset of 3074 individuals. Sub-haplotypes were phased and estimated as described above, and frequencies for each H1.1, H1.2 and H1.3 sub-haplotype were determined.

### Human brain expression analysis

Publicly available RNA-seq expression data from human postmortem dorsolateral prefrontal (PFC) and temporal (TCX) cortices (Table S5) and associated genotype data were obtained from Synapse (synapse.org; The Religious Orders Study and Memory and Aging Project (ROSMAP) syn3219045; MayoRNAseq syn5550404; CommonMind Consortium syn2759792). Genotype data for chromosome 17 underwent the same QC and imputation pipeline as described above. Data were stratified by 17q21.31 H1/H2 haplotype using the H2 tag SNP rs8070723. For sub-haplotype analysis, blocks previously defined in the PD analysis were applied to the genotype data and haplotypes were estimated in the same manner. Statistical analysis was carried out in R version 3.4.0. For analysis of *MAPT* splicing, percent spliced in (PSI) values were generated for exons 2, 3 and 10 using Mixture of Isoforms (MISO) [62]. Gene expression and PSI residuals were generated by linear regression using sex, age of death, post-mortem interval and RNA integrity score as covariates. The resulting residuals were then transformed into z-scores and combined across datasets. Statistical differences in gene expression between genotypes and sub-haplotypes were determined by linear regression applied to the adjusted and combined z-scores.

### dPCR

Human genomic DNA and accompanying genotype data was kindly provided by Drs. Raj, Crary and Charney (Mount Sinai School of Medicine, NY) and by the Alzheimer’s Disease Research Center (ADRC; Table S5). Sub-haplotypes were called from these genotype data in the same manner as described above. To examine copy number variation in the 17q21.31 locus, digital PCR was carried out using the ThermoFisher QuantStudio 3D digital PCR chip system. Taqman dPCR probes for loci within the alpha, beta and gamma CNV regions [20], as well as within *LRRC37A* and *MAPT* were selected for analysis (Table S7).

### Cell lines

Human induced pluripotent stem cells (iPSCs) were obtained from the Knight Alzheimer’s Disease Research Center at Washington University [63], the NIH Childhood-onset Schizophrenia study [64], the New York Stem Cell Foundation (NYSCF) and the New South Wales Brain Bank (NSWBB) (Table S8). The Icahn School of Medicine at Mount Sinai IRB reviewed the relevant operating protocols as well as this specific study and determined it was exempt from approval.

### Cell culture

Unless otherwise specified, all cell culture materials were obtained from ThermoFisher Scientific. Human embryonic kidney cells (HEK293T) were cultured in Dulbecco's Modified Eagle Medium/F-12 with HEPES, supplemented with 10% fetal bovine serum (FBS) and 1% Penicillin–Streptomycin. Cells were passaged every 3–4 days using Trypsin–EDTA (0.25%). For *LRRC37A*/2 overexpression experiments, HEK293T cells were seeded at a density of 1.4 × 10^5^ cells per well in 6-well plates and transfected with 0.5–2.5ug of *LRRC37A* plasmid (Origene) or empty vector control (Origene) using Lipofectamine 3000. Cells were harvested 48 h after transfection.

For qRTPCR, protein biochemistry and *LRRC37A2* knockdown experiments, iPSC lines (Table S8) were maintained in complete StemFlex media supplemented with 1% penicillin/streptomycin on Matrigel (BD biosciences), and were differentiated to neural progenitor cells (NPCs) as previously described [65]. Forebrain neuron-enriched cultures and astrocyte cultures were differentiated from NPCs as previously described [65, 66]. Neuronal and astrocytic identity was confirmed by immunofluorescence for common neuronal and astrocytic markers (MAP2 (Abcam), Tuj1 (Cell Signaling Technologies), S100β (Sigma Aldrich) and EAAT1 (Abcam).

Genomic DNA was extracted using the DNeasy Blood and Tissue kit (Qiagen) and underwent genotyping with Taqman assays for H2 tag SNPs rs8070723 and rs1052553 in order to confirm 17q21.31 haplotype.

### LRRC37A2 knockdown

iPSC-derived astrocytes from four donors were seeded at low density in a 24-well plate, and were infected with either *LRRC37A2* shRNA viral particles (Santa Cruz) or scrambled control viral particles (Origene) with an MOI = 10 for 24 h. Infected cells were then washed with fresh media, then maintained and expanded as previously described [67]. *LRRC37A2* knockdown was confirmed by qRT-PCR using commercially available Taqman probes, and was normalized to *GAPDH* expression.

### Scratch wound assay

LRRC37A2 and scrambled control shRNA-treated astrocytes were seeded at high density into IncuCyte ImageLock plates coated with Matrigel (Corning). Upon reaching confluency, scratch wounds were applied to each well using the Incucyte WoundMaker tool, and cell culture media was replaced in order to remove dead cells and debris. Astrocytes were then either treated with 200 ng/ml monomeric recombinant α-synuclein (Abcam) or the equivalent volume of PBS. Cells were then imaged every hour for 50 h in the IncuCyte live cell imaging platform. Images were analyzed using the IncuCyte software with Scratch Wound module for automated detection of wound confluency and cell density.

### Immunofluorescence and image analysis

iPSC-derived astrocytes were grown in 96-well plates and fixed with 10% Formalin (Sigma Aldrich) for 15 min at room temperature, followed by 3 × washes with PBS. Fixed cells were permeabilized with 0.1% Triton x-100 and blocked with 1% BSA in PBS for 30 min at room temperature. Cells were incubated with antibodies against either IL-16 or IL-32 (both Abcam) overnight at 4 °C, washed × 3 with PBS and incubated with secondary antibodies (AlexaFluor anti-rabbit 647, ThermoFisher Scientfic) at 1:100 for 2 h at room temperature. Cells were counterstained with DAPI for 10 min at room temperature, then washed × 3 with PBS. Labeled astrocytes underwent automated imaging on the ThermoFisher Cellnsight CX7 High Content Screening platform with a 40 × objective and widefield imaging. Exposure times for IL-16 and IL-32 were manually fixed across all fields and wells. Sixteen images per cell line were arbitrarily taken per condition. Image intensity analysis was carried out using ImageJ v1.53 s. A minimum grey intensity threshold was set to enable cell detection by positive pixels, and mean grey intensity was determined across positive pixels per field of view. qRT-PCR.

RNA was extracted from HEK293T cells, iPSC-derived neurons, astrocytes and human brain tissue using the RNeasy Mini kit (Qiagen) and reverse transcribed using the High-Capacity RNA-to-cDNA kit (ThermoFisher Scientific). Gene expression was measured by commercially available Taqman qRTPCR assays.

### RNA-seq

RNA was prepared as described above. Library preparation with poly-A selection and sequencing with 150 base pair paired-end reads was carried out at Genewiz. Sequenced reads were trimmed for Illumina TruSeq adapters, and quantified for gene expression values in TPM (Transcripts Per Kilobase Million) using Salmon [68] guided by the GENCODE human transcriptome model (GRCh38 version 28, Ensembl 92). TPM data was imported into the R (version 3.5.1) programming environment for visualization and analysis, and differential expression of *LRRC37A*/2 overexpression compared to the control was analyzed using the moderated t-test implemented in limma [69]. Differentially expressed genes (DEGs) were defined by ±  ≥ 1.5 expression fold change and adjusted *p* < 0.05. Gene set enrichment analysis was performed with the Broad Institute’s MSigDB annotations [70]. Analysis of GO enrichment terms was carried out using g:Profiler (https://biit.cs.ut.ee/gprofiler/gost) [71, 72] and visualized using Cytoscape v3.7.1 [73] with the EnrichmentMap [74] plugin. Additional pathway analyses were carried out using Ingenuity Pathway Analysis (QIAGEN Inc., https://www.qiagenbioinformatics.com/products/ingenuitypathway-analysis) using genes with a fold change ±  ≥ 1.

### Protein biochemistry

Membrane and cytosolic proteins were isolated from HEK293T cells, iPSC-derived neurons and iPSC-derived astrocytes using the MEM-PER Plus Membrane Protein Extraction Kit (ThermoFisher Scientific), and protein concentrations were determined by bicinchoninic acid (BCA) assay (ThermoFisher Scientific). For western blotting, protein fractions were subject to SDS-PAGE electrophoresis through BOLT Bis–Tris gels (ThermoFisher Scientific) and were blotted onto nitrocellulose membranes. Membrane fractions were confirmed by labelling with an anti-pan-Cadherin antibody (Cell Signaling Technology), and cytosolic fractions were confirmed by labelling with anti-HSP90 (Cell Signaling Technology). Membranes were stripped using Restore plus western blot stripping buffer and re-probed with an anti-LRRC37A antibody (ThermoFisher Scientific).

### Single nuclei sequencing data analysis

PD midbrain snuc-seq gene counts and cell metadata34 were downloaded from GEO (GSE157783). AD PFC snuc-seq filtered gene counts and cell metadata33 were downloaded from Synapse (syn18681734). Data underwent filtering and processing using Seurat v4.075,76. Briefly, for PD midbrain data, cells expressing fewer than 200 genes, with fewer than 2500 reads and with greater than 10% mitochondrial gene expression were removed. Count data was normalized using SCTransform77, while regressing out mitochondrial rate, number of expressed genes and the number of reads per cell. Principal components analysis was carried out using the top 3000 most variable genes, and data reduction was performed with UMAP78. Cell types were determined using the provided annotations on GEO. Astrocytes were then isolated and re-scaled. Differential gene expression between PD and control cells was carried out on the raw count data using the MAST model with percent mitochondrial gene expression as a covariate. For AD PFC data, cells with fewer than 2000 reads, expressing fewer than 200 genes and abnormally high ratios of counts mapped to mitochondrial genes relative to the total number of detected genes were removed. Count data was normalized using the SCANPY package79 and principal components analysis and data reduction was carried out as described above. Cell types were determined using the annotations provided in the cell metadata, and genotypes were determined using genotype data from the ROSMAP cohort as described above. Differential gene expression between haplotypes was carried out on the raw count data using the MAST model with pathological diagnosis, sex and age at death as covariates.

### OPAL multiplex labelling

Formalin fixed paraffin embedded substantia nigra sections from human controls (*N* = 4), PSP (*N* = 5) and PD (*N* = 5) cases were acquired from the Mount Sinai Neuropathology Brain Bank, with neuropathological diagnosis being determined by Dr. John Crary. All post-mortem tissues were collected in accordance with the relevant guidelines and regulations at the Icahn School of Medicine at Mount Sinai. Multiplexed immunofluorescent staining was carried out on 4-6 µm sections using the Opal Polaris 7 color IHC detection kit (Akoya biosciences) according to manufacturer’s instructions. Briefly, slides were baked for 1 h at 65 °C, then deparaffinized with xylene and rehydrated with a graded series of ethanol concentrations. For epitope retrieval, slides were microwaved in AR buffer for 45 s at 100% power, followed by an additional 15 min at 20% power. After cooling, slides were blocked for 10 min in blocking buffer then incubated with the first primary antibody at room temperature for 30 min. Slides were rinsed three times in TBS-T, then incubated with the secondary polymer HRP for 1 h at room temperature. After additional washes, the first Opal fluorophore was incubated with the slides for 10 min at room temperature, followed by further washes in TBS-T. This process was repeated from the microwave treatment step for each additional primary antibody, followed by one final repetition of the microwave treatment to strip the primary-secondary antibody complex from the tissue. Once all primary antibodies had been introduced, slides were counterstained with DAPI for 5 min at room temperature, washed with TBS-T and coverslips were mounted using ProLong Diamond Antifade mounting reagent (ThermoFisher Scientific). Multispectral imaging was carried out using the Vectra Quantitative Pathology Imaging system, applying quantitative unmixing of fluorophores and removal of tissue autofluorescence. Images were visualized using the HALO image analysis platform (Indica Labs).

### Co-Immunoprecipitation

Frozen substantia nigra tissue was selected from the same Control (*N* = 3), PSP (*N* = 3) and PD (*N* = 3) cases used for OPAL multiplex immunofluorescence. Protein lysates were generated using cell lysis buffer (NEB) and brief sonication on ice, followed by centrifugation to pellet insoluble material. Co-immunoprecipitation was carried out using the Dynabeads Protein G immunoprecipitation kit (ThermoFisher Scientific), with an anti-α-synuclein antibody (Abcam) as bait. Proteins bound to beads were eluted and assayed by western blot (as described above) and probed with an anti-LRRC37A antibody (ThermoFisher Scientific). Whole protein lysate and IgG only controls were run on the same blots.

## Supplementary Information

**Additional file 1. **Supplementary fig 1

**Additional file 2. **Supplementary fig 2

**Additional file 3. **Supplementary fig 3

**Additional file 4. **Supplementary fig 4

**Additional file 5. **Supplementary fig 5

**Additional file 6. **Supplementary fig 6

**Additional file 7. **Supplementary fig 7 

**Additional file 8. **Supplementary table1

**Additional file 9. **Supplementary table 2

**Additional file 10. **Supplementary table 3

**Additional file 11. **Supplementary table 4

**Additional file 12. **Supplementary table 5

**Additional file 13. **Supplementary table 6

**Additional file 14. **Supplementary table 7

**Additional file 15. **Supplementary table 8

## Data Availability

All aligned read counts and FASTQ files for *LRRC37A*-overexpressing HEK293T cells will be deposited to the Gene Expression Omnibus once the manuscript is accepted for publication.

## Abbreviations

PD: Parkinson’s disease
AD: Alzheimer’s disease
CBD: Corticobasal degeneration
PSP: Progressive supranuclear palsy
dPCR: Digital polymerase chain reaction
qRT-PCR: Quantitative reverse transcription polymerase chain reaction
DNA: Deoxyribonucleic acid
RNA-seq: Ribonucleic acid sequencing
LD: Linkage disequilibrium
Mb: Megabase
OR: Odds ratio
SNP: Single nucleotide polymorphism
CI: Confidence interval
CNV: Copy number variation
GO: Gene ontology
IPA: Ingenuity pathway analysis
iPSC: Induced pluripotent stem cell
GWAS: Genome-wide association study
IgG: Immunoglobulin G
AMP-AD: Accelerating Medicines Partnership Alzheimer’s disease
PFC: Prefrontal cortex
TCX: Temporal cortex
MAPT: Microtubule associated protein tau
LRRC37A/2: Leucine rich repeat containing 37A and 37A2
KANSL1: KAT8 regulatory NSL complex subunit 1
IL17F: Interleukin 17F
IL32: Interleukin 32
IL16: Interleukin 16
CD4: Cluster of differentiation 4
GFAP: Glial fibrillary acidic protein
IBA1: Ionized calcium binding adaptor molecule 1
GBA: Glucosylceramidase beta
LRRK2: Leucine rich repeat kinase 2
PINK1: PTEN induced kinase 1
TGFA: Transforming growth factor alpha
GDNF: Glial cell line-derived neurotrophic factor
IPDGC: International parkinson’s disease genetics consortium
NIA: National institute of aging
GER: German
FIN: Finnish
NL: Netherlands
SP: Spanish
QC: Quality control
MAF: Minor allele frequency
YRI: Yoruba
CEU: Northern Europeans from Utah
CHB: Han Chinese in Beijing
HRC: Haplotype reference consortium
SVS: SNP variation suite
EM: Expectation-maximization
PSI: Percent spliced in
MISO: Mixture of isoforms
ADRC: Alzheimer’s disease research center
IRB: Institutional review board
FBS: Fetal bovine serum
NPC: Neural progenitor cell
TPM: Transcripts per million
DEG: Differentially expressed gene
BCA: Bicinchoninic acid
SDS-PAGE: Sodium dodecyl sulfate polyacrylamide gel electrophoresis
IHC: Immunohistochemistry
AR: Antigen retrieval
HRP: Horseradish peroxidase
TBS-T: Tris-buffered saline with tween

## Declarations

### Declarations

**Ethical Approval and Consent to Participate**

Not applicable.
