# Supplementary figures and images for: 17q21.31 sub-haplotypes underlying H1-associated risk for Parkinson’s disease are associated with LRRC37A/2 expression in astrocytes
Source: Mol Neurodegener. 2022 Jul 15;17:48. doi: 10.1186/s13024-022-00551-x (PMC9284779; doi:10.1186/s13024-022-00551-x)

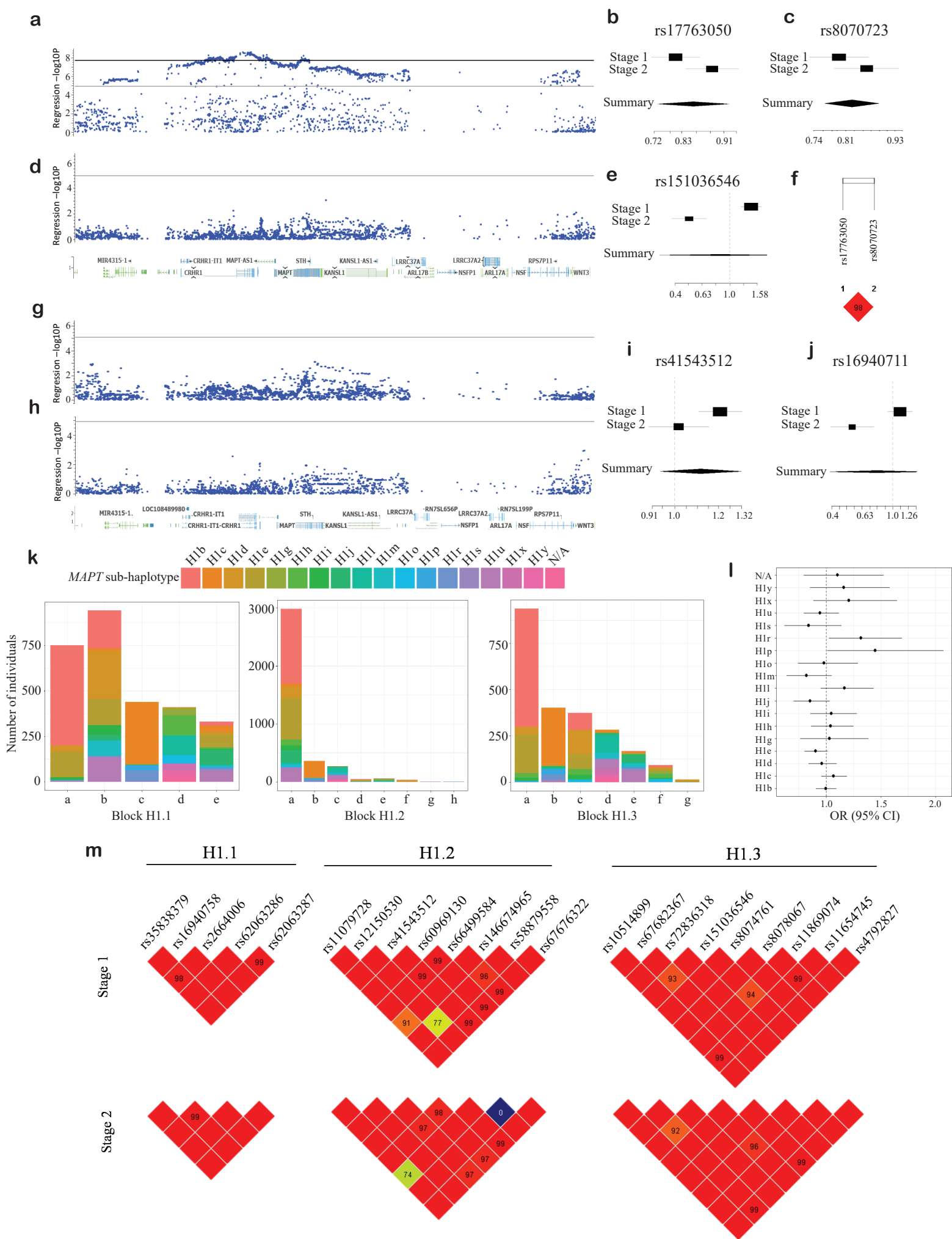

Supplement: Supplementary file 1 — Additional file 1. Supplementary fig 1 [file 13024_2022_551_MOESM1_ESM.pdf]

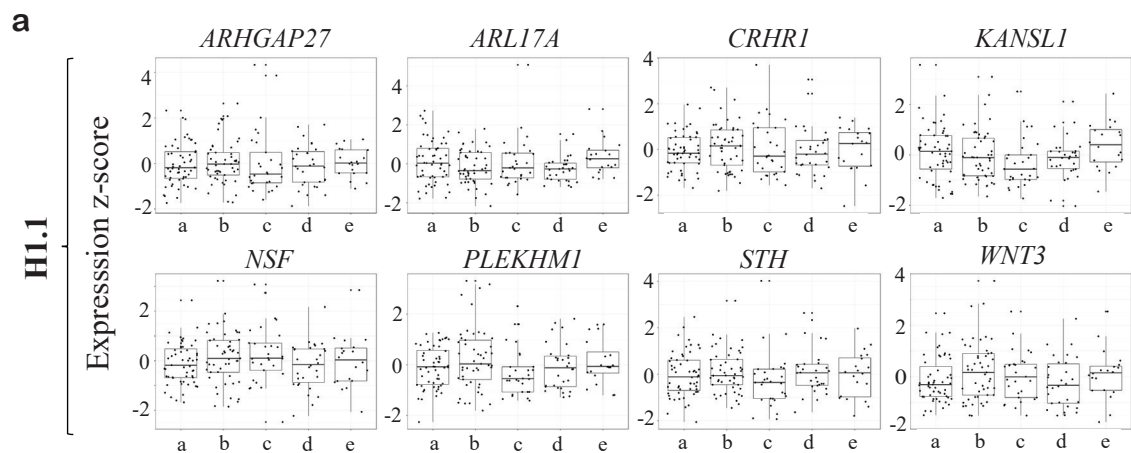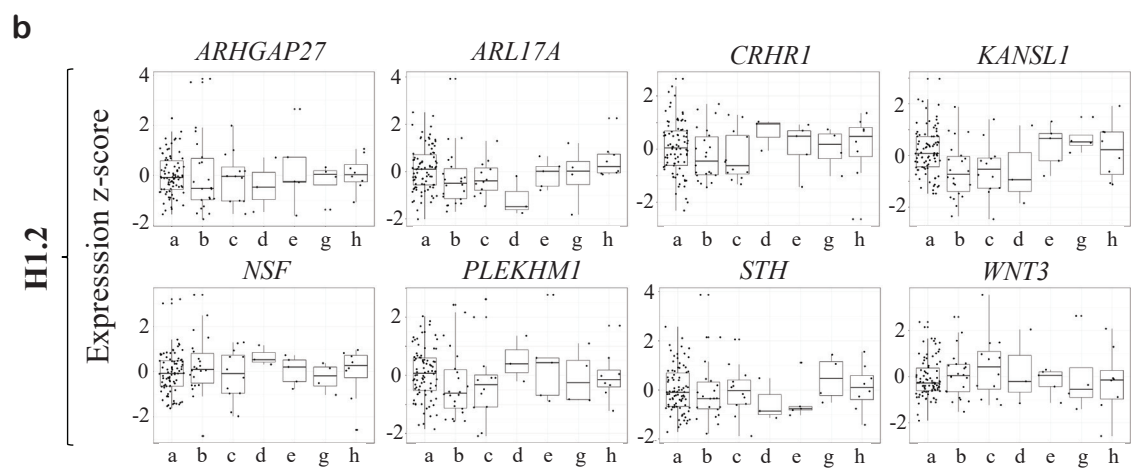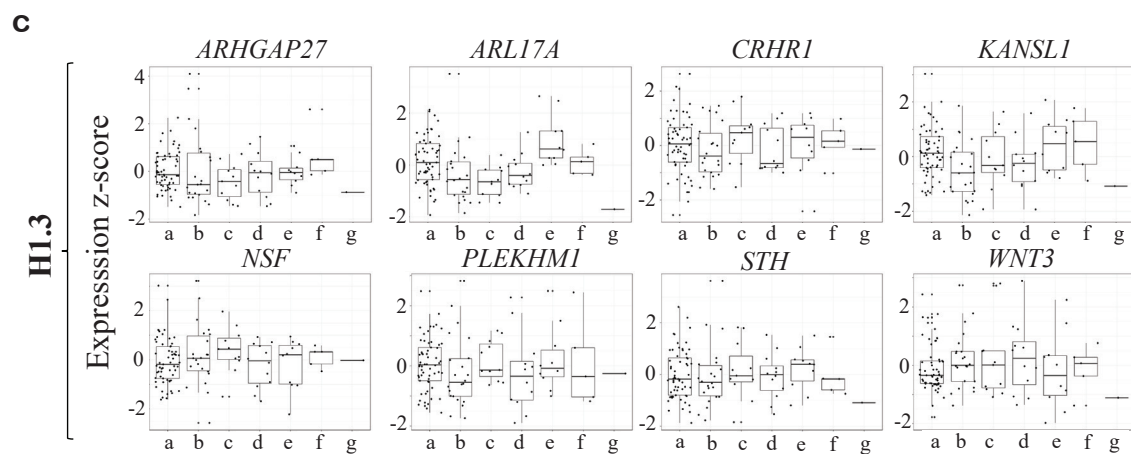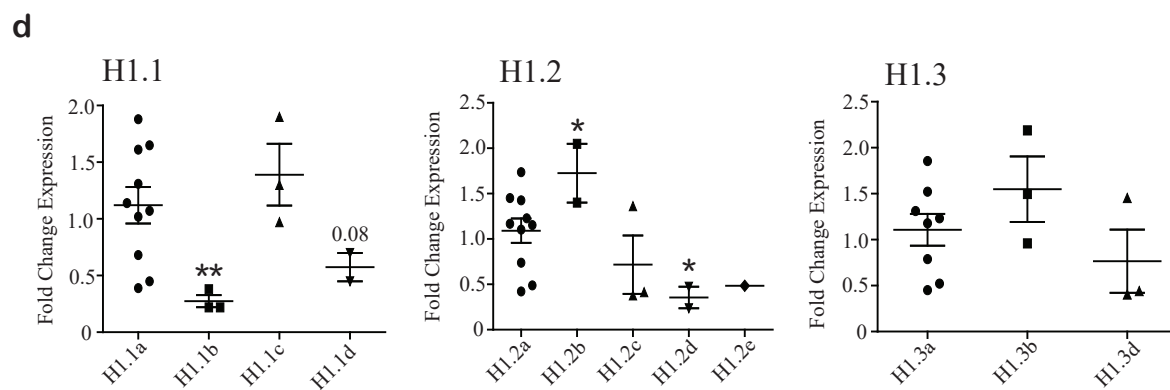

Supplement: Supplementary file 3 — Additional file 3. Supplementary fig 3 [file 13024_2022_551_MOESM3_ESM.pdf]

**a**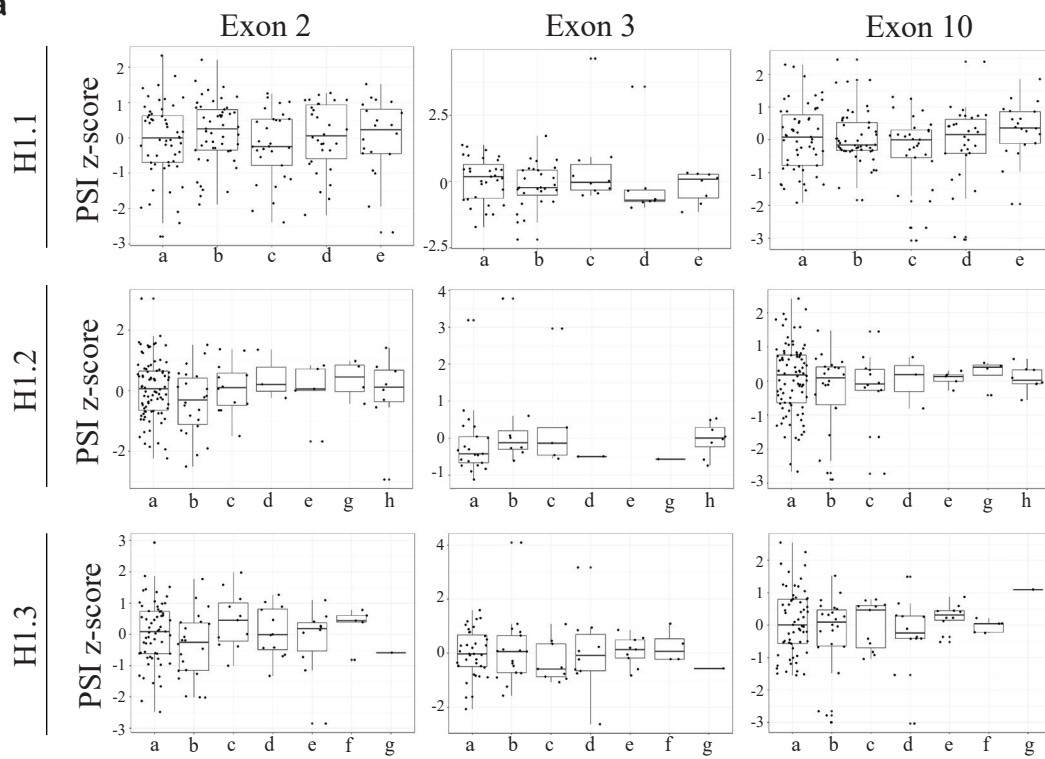**b**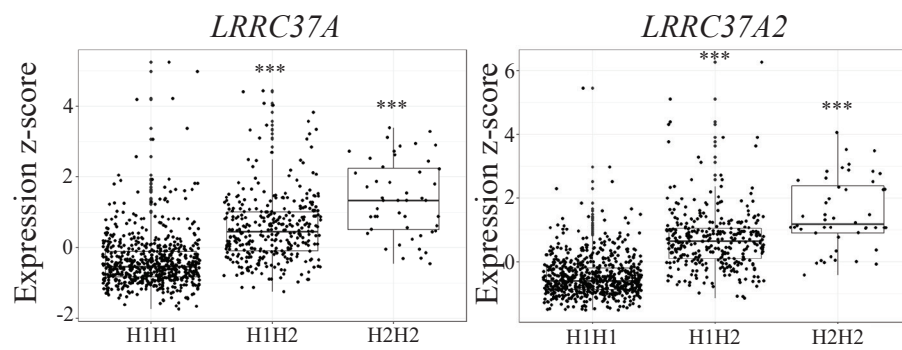

Supplement: Supplementary file 4 — Additional file 4. Supplementary fig 4 [file 13024_2022_551_MOESM4_ESM.pdf]

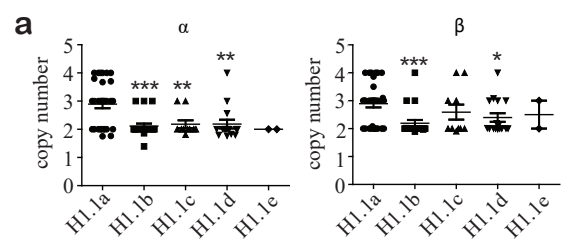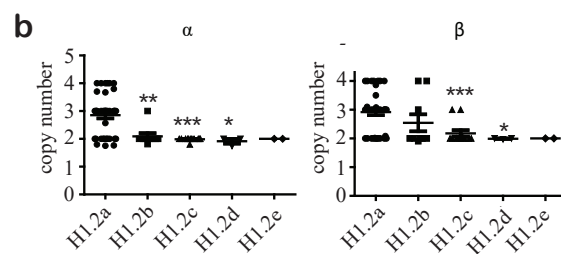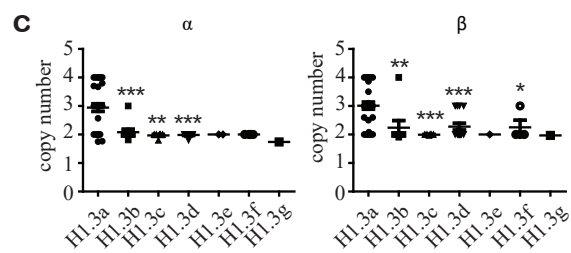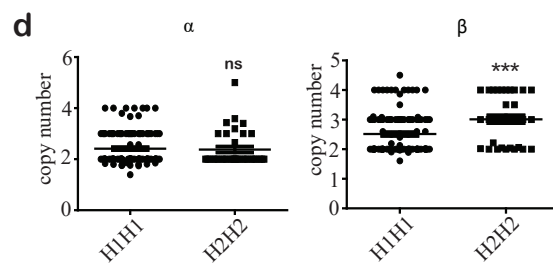

Supplement: Supplementary file 5 — Additional file 5. Supplementary fig 5 [file 13024_2022_551_MOESM5_ESM.pdf]

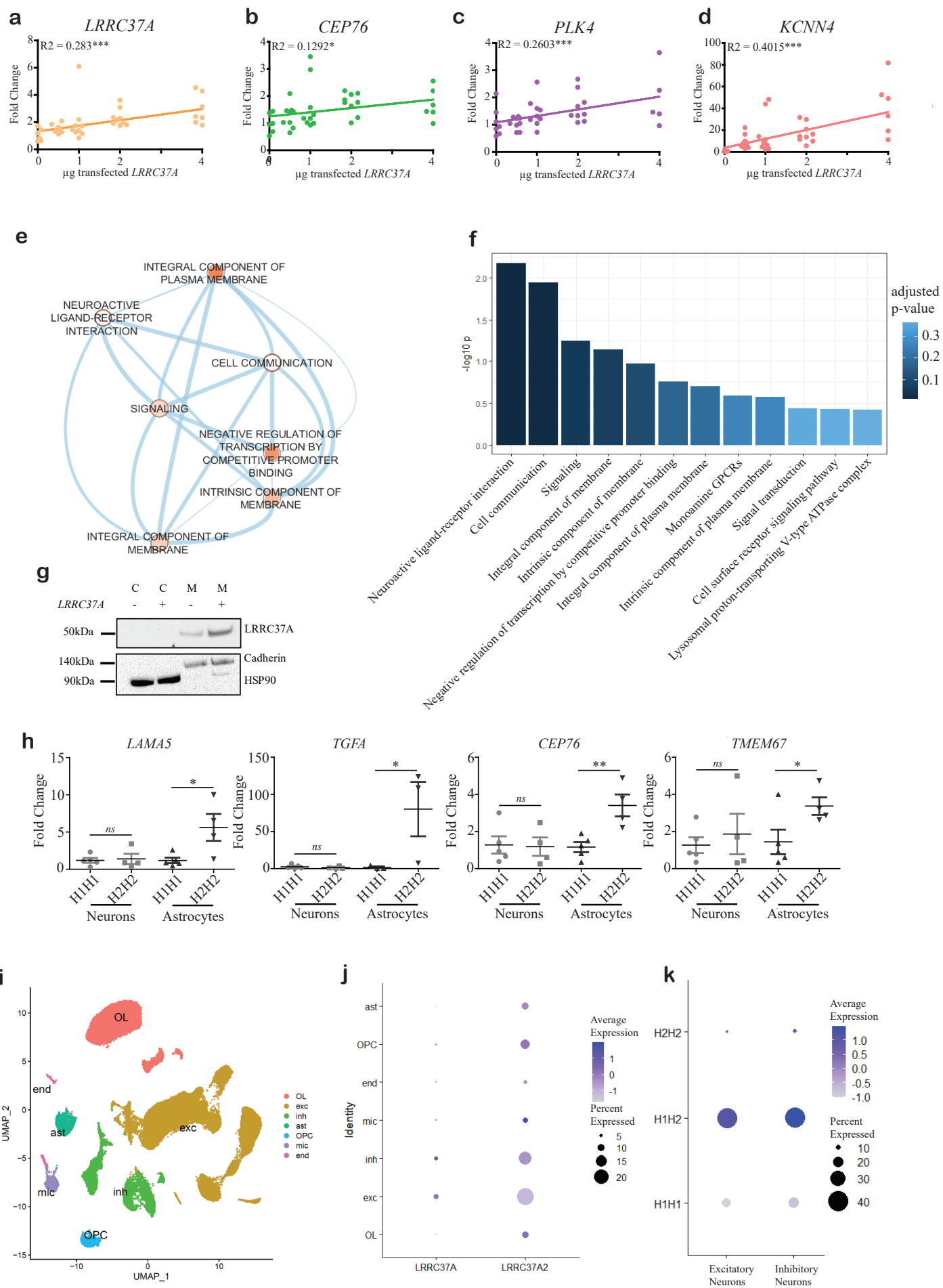

Supplement: Supplementary file 6 — Additional file 6. Supplementary fig 6 [file 13024_2022_551_MOESM6_ESM.pdf]

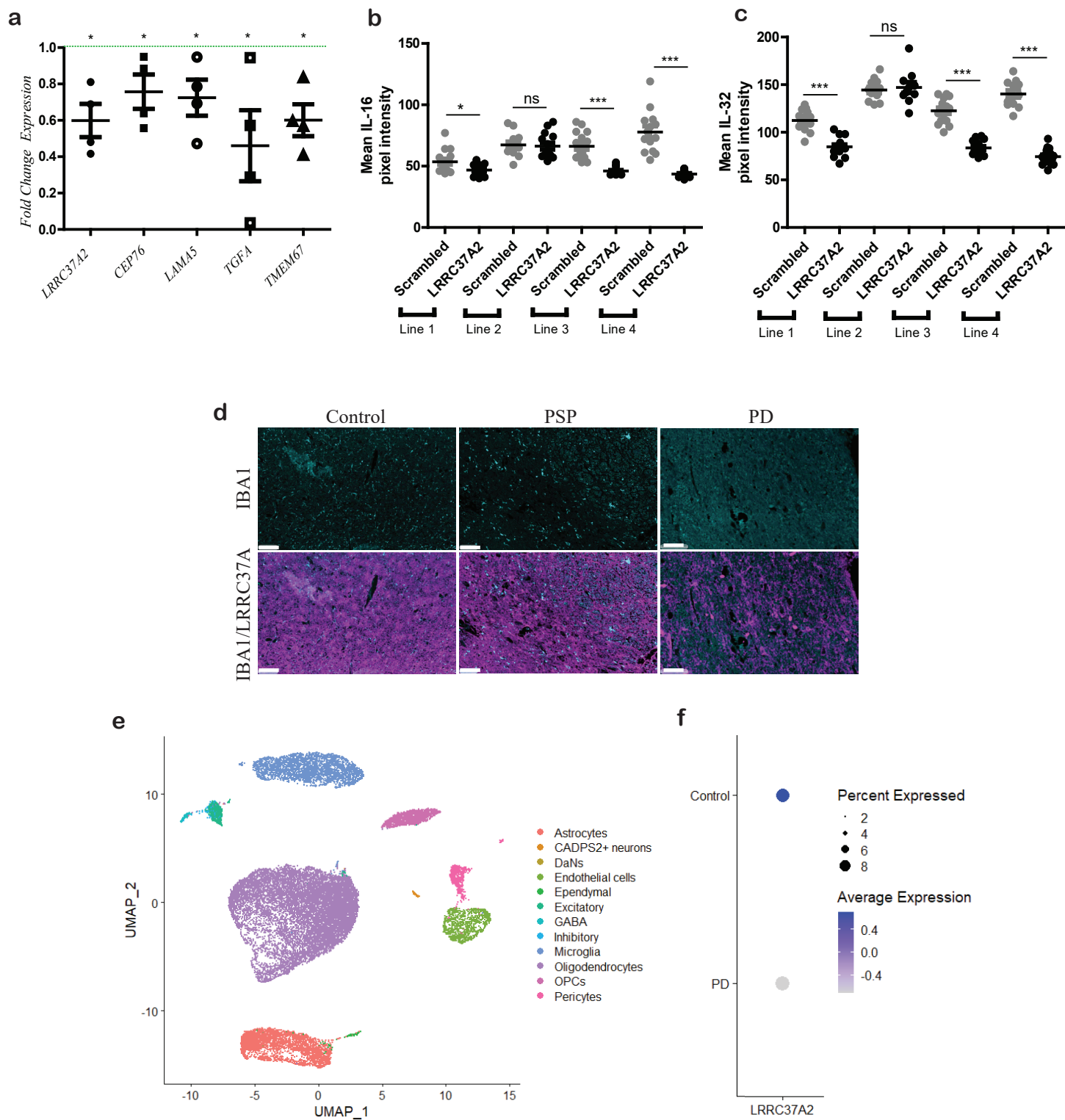

Supplement: Supplementary file 7 — Additional file 7. Supplementary fig 7 [file 13024_2022_551_MOESM7_ESM.pdf]
