# Supplementary material for: 17q21.31 sub-haplotypes underlying H1-associated risk for Parkinson’s disease are associated with LRRC37A/2 expression in astrocytes
Source: Mol Neurodegener. 2022 Jul 15;17:48. doi: 10.1186/s13024-022-00551-x (PMC9284779; doi:10.1186/s13024-022-00551-x)
Supplement: Supplementary file 2 — Additional file 2. Supplementary fig 2 [file 13024_2022_551_MOESM2_ESM.pdf]

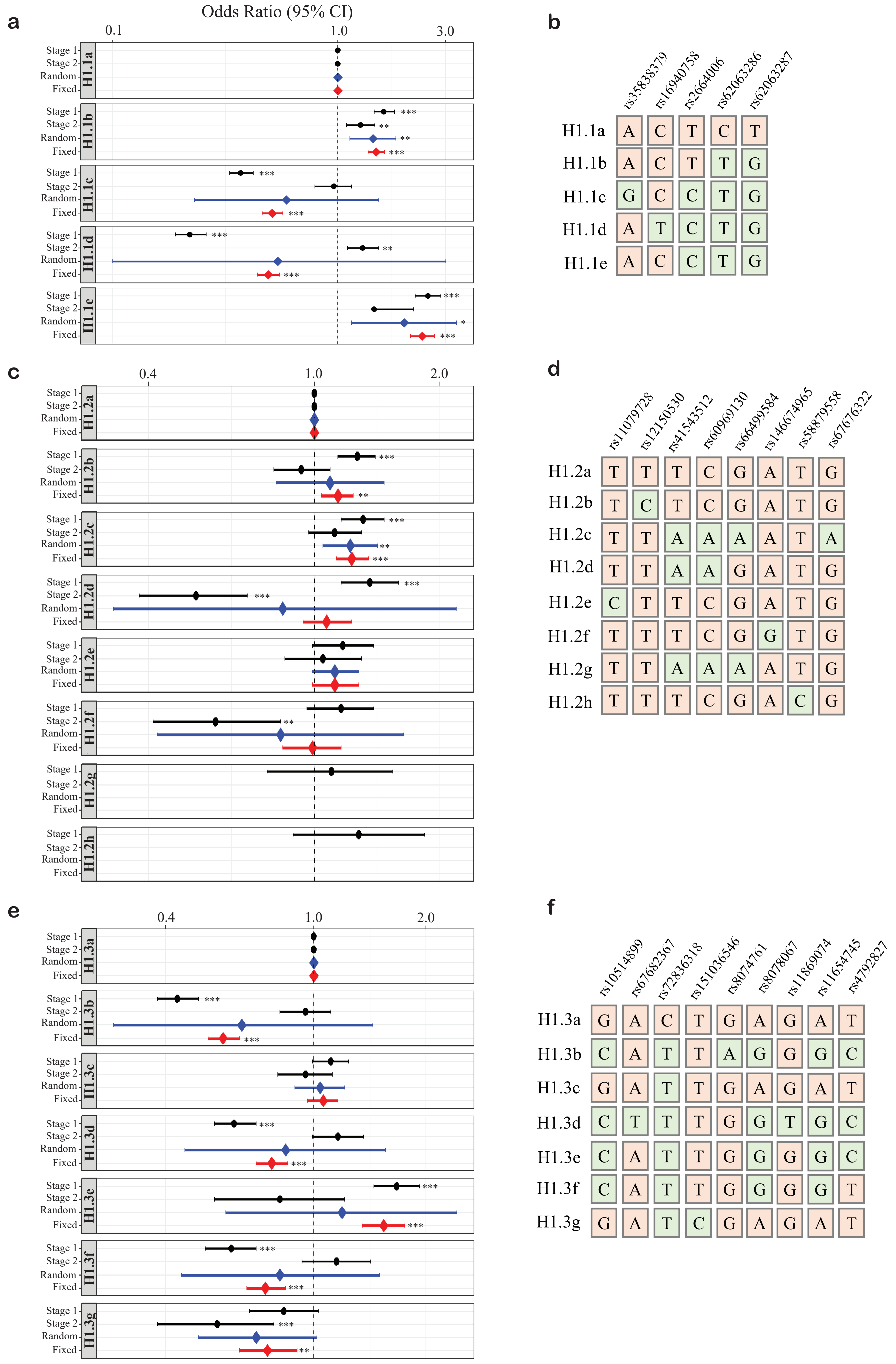
a
Odds Ratio (95% CI)
0.1
1.0
3.0
H1.1a
H1.1b
H1.1c
H1.1d
H1.1e
Stage 1
Stage 2
Random
Fixed
b
rs35838379
rs16940758
rs2664006
rs62063286
rs62063287
H1.1a A C T C T
H1.1b A C T T G
H1.1c G C C T G
H1.1d A T C T G
H1.1e A C C T G
c
0.4
1.0
2.0
H1.2a
H1.2b
H1.2c
H1.2d
H1.2e
H1.2f
H1.2g
H1.2h
d
rs11079728
rs12150530
rs41543512
rs60969130
rs66499584
rs146674965
rs58879558
rs67676322
H1.2a T T T C G A T G
H1.2b T C T C G A T G
H1.2c T T A A A A T A
H1.2d T T A A G A T G
H1.2e C T T C G A T G
H1.2f T T T C G G T G
H1.2g T T A A A A T G
H1.2h T T T C G A C G
e
0.4
1.0
2.0
H1.3a
H1.3b
H1.3c
H1.3d
H1.3e
H1.3f
H1.3g
f
rs10514899
rs67682367
rs72836318
rs151036546
rs8074761
rs8078067
rs11869074
rs11654745
rs4792827
H1.3a G A C T G A G A T
H1.3b C A T T A G G G C
H1.3c G A T T G A G A T
H1.3d C T T T G G T G C
H1.3e C A T T G G G G C
H1.3f C A T T G G G G T
H1.3g G A T C G A G A T
