# Supplementary material for: 17q21.31 sub-haplotypes underlying H1-associated risk for Parkinson’s disease are associated with LRRC37A/2 expression in astrocytes
Source: Mol Neurodegener. 2022 Jul 15;17:48. doi: 10.1186/s13024-022-00551-x (PMC9284779; doi:10.1186/s13024-022-00551-x)
Supplement: Supplementary file 8 — Additional file 8. Supplementary table1 [file 13024_2022_551_MOESM8_ESM.docx]

**Table S1. Top SNPs in the 17q21.31 locus associated with PD in complete data and H1 homozygote analyses**

| **All data** |  |  | |  |  |  |  |  |  |  |  |
| --- | --- | --- | --- | --- | --- | --- | --- | --- | --- | --- | --- |
|  | Stage 1 Top SNP | | Position | *p*-value | -log_10_ *p*-value | FDR *p*-value | Major Allele | Minor Allele | MAF (case/control) | OR (95% CI) | OR fisher *p*-value |
| Stage 1 | rs17763050 | 17:43903336 | | 2.74E-09 | 8.562 | 5.44E-04 | G | A | 0.21 (0.17/0.22) | 0.75 (0.69-0.81) | 8.50E-13 |
| Stage 2 |  |  |  | 6.10E-01 | 0.22 | 9.70E-01 |  |  | 0.26 (0.25/0.28) | 0.87 (0.8-0.96) | 3.77E-03 |
| Meta |  |  |  | - | - | - |  |  | - | 0.82 (0.73-0.93) | 1.42E-03^Ŧ^ |
|  |  |  | |  |  |  |  |  |  |  |  |
|  | Stage 2 Top SNP | Position | | p-value | -log_10_ *p*-value | FDR *p*-value | Major Allele | Minor Allele | MAF (case/control) | OR (95% CI) | OR fisher *p*-value |
| Stage 1 | rs151036546 | 17:44122049 | | 2.59E-03 | 2.59 | 1.57E-01 | T | C | 0.03 (0.04/0.03) | 1.42 (1.12-1.7) | 1.13E-04 |
| Stage 2 |  |  |  | 8.7E10-04 | 3.06 | 2.70E-01 |  |  | 0.02 (0.01/0.03) | 0.51 (0.37-0.68) | 2.72E-06 |
| Meta |  |  |  | - | - | - |  |  | - | 0.85 (0.31-2.35) | 7.6E-01^Ŧ^ |
|  |  |  | |  |  |  |  |  |  |  |  |
|  | H2 Tag SNP | Position | | p-value | -log_10_ *p*-value | FDR *p*-value | Major Allele | Minor Allele | MAF (case/control) | OR (95% CI) | OR fisher *p*-value |
| Stage 1 | rs8070723 | 17:44081064 | | 1.70E-07 | 6.77 | 1.96E-05 | A | G | 0.2 (0.16/0.21) | 0.77 (0.71-0.83) | 1.66E-10 |
| Stage 2 |  |  |  | 4.70E-01 | 0.33 | 9.50E-01 |  |  | 0.25 (0.24/0.27) | 0.86 (0.78-0.94) | 1.07E-03 |
| Meta |  |  |  | - | - | - |  |  | - | 0.82 (0.76-0.89) | 6.32E-07^Ŧ^ |
|  |  |  | |  |  |  |  |  |  |  |  |
| **H1 homozygotes only** | |  | |  |  |  |  |  |  |  |  |
|  | Stage 1 Top SNP | Position | | *p*-value | -log_10_ *p*-value | FDR *p*-value | Major Allele | Minor Allele | MAF (case/control) | OR (95% CI) | OR fisher *p*-value |
| Stage 1 | rs41543512 | 17:44090778 | | 8.4E10-4 | 3.08 | 0.55 | T | A | 0.23 (0.25/0.21) | 1.21 (1.10-1.32) | 4.44E-05 |
| Stage 2 |  |  |  | 0.61 | 0.22 | 0.99 |  |  | 0.26 (0.24/0.24) | 1.02 (0.89-1.16) | 0.822 |
| Meta |  |  |  | - | - | - |  |  | - | 1.11 (0.94-1.32) | 0.21^Ŧ^ |
|  |  |  | |  |  |  |  |  |  |  |  |
|  | Stage 2 Top SNP | Position | | *p*-value | -log_10_ *p*-value | FDR *p*-value | Major Allele | Minor Allele | MAF (case/control) | OR (95% CI) | OR fisher *p*-value |
| Stage 1 | rs139217062 | 17:43849514 | | - | - | - | T | C | - | - | - |
| Stage 2 |  |  |  | 2.80E-03 | 2.55 | 0.66 |  |  | 0.02 (0.03/0.02) | 1.82 (1.25-2.69) | 1.01E-02 |
| Meta |  |  |  | - | - | - |  |  | - | - | - |
|  |  |  | |  |  |  |  |  |  |  |  |
|  | Stage 2 2nd SNP | Position | | *p*-value | -log_10_ *p*-value | FDR *p*-value | Major Allele | Minor Allele | MAF (case/control) | OR (95% CI) | OR fisher *p*-value |
| Stage 1 | rs16940711 | 17:43930006 | | 0.2 | 0.7 | 0.94 | A | C | 0.05 (0.05/0.04) | 1.11 (0.92-1.34) | 0.26 |
| Stage 2 |  |  |  | 8.80E-03 | 2.06 | 0.79 |  |  | 0.03 (0.02/0.04) | 0.54 (0.39-0.76) | 2.51E-04 |
| Meta |  |  |  | - | - | - |  |  | - | 0.79 (0.39-1.59) | 0.5^Ŧ^ |

**Ŧ** Random effects meta-analysis; **FDR** = False discovery rate; **MAF** = Minor allele frequency, **OR** = Odds ratio; **CI** = Confidence interval
