# Supplementary material for: 17q21.31 sub-haplotypes underlying H1-associated risk for Parkinson’s disease are associated with LRRC37A/2 expression in astrocytes
Source: Mol Neurodegener. 2022 Jul 15;17:48. doi: 10.1186/s13024-022-00551-x (PMC9284779; doi:10.1186/s13024-022-00551-x)
Supplement: Supplementary file 9 — Additional file 9. Supplementary table 2 [file 13024_2022_551_MOESM9_ESM.docx]

**Table S2. Data summary for Stage 1 and Stage 2 PD analyses**

|  | **Cohort** | **Array** | **Total N (Case/Control)** | **H1H1 (Case/Control)** | **H2H2 (Case/Control)** | **#SNPs used for imputation (Chr17)** | **#Imputed SNPs (Chr17) R2 >= 0.3** |
| --- | --- | --- | --- | --- | --- | --- | --- |
| Stage 1 | NIH (IPDGC) | Illumina 550K v1 & v3 | 3906 (905/3001) | 2447 (606/1841) | 161 (31/130) | 11941 | 634666 |
|  | NL (IPDGC) | Illumina 550K & 610K | 2725 (767/1958) | 1654 (500/1154) | 144 (39/105) | 13457 | 530635 |
|  | FIN (IPDGC) | Illumina 370K | 860 (368/492) | 732 (316/416) | 7 (4/3) | 7970 | 357255 |
|  | GER (IPDGC) | Illumina 550K v1 | 1673 (740/933) | 1121 (527/594) | 51 (19/32) | 11803 | 481302 |
|  | Merged | - | 9164 (2780/6384) | 5932 (1937/3995) | 378 (94/284) | - | 295782 |
|  |  |  |  |  |  |  |  |
| Stage 2 | McGill (IPDGC) | Illumina Omini Express & NeuroChip v.1.0 | 1485 (582/903) | 859 (354/505) | 70 (19/51) | 17467 | 515155 |
|  | SP (IPDGC) | NeuroChip v.1.0 & v.1.1 | 3444 (2117/1327) | 1861 (1184/677) | 243 (130/113) | 16590 | 493744 |
|  | Merged | - | 4929 (2699/2230) | 2925 (1538/1182) | 313 (149/164) | - | 368729 |
