# Supplementary material for: 17q21.31 sub-haplotypes underlying H1-associated risk for Parkinson’s disease are associated with LRRC37A/2 expression in astrocytes
Source: Mol Neurodegener. 2022 Jul 15;17:48. doi: 10.1186/s13024-022-00551-x (PMC9284779; doi:10.1186/s13024-022-00551-x)
Supplement: Supplementary file 10 — Additional file 10. Supplementary table 3 [file 13024_2022_551_MOESM10_ESM.docx]

| **Table S3. *MAPT* sub-haplotype association with PD risk in Stage 1 data** | | | |
| --- | --- | --- | --- |
|  |  |  |  |
| **Haplotype** | **Frequency (Case/Control)** | **OR (95% CI)** | **Regression -log10 p** |
| H1b | 0.21 (0.21/0.21) | 0.99 (0.91-1.1) | 0.19 |
| H1c | 0.14 (0.14/0.15) | 1.06 (0.95-1.12) | 0.49 |
| H1d | 0.09 (0.09/0.09) | 0.96 (0.84-1.10) | 0.44 |
| H1e | 0.13 (0.12/0.14) | 0.90 (0.8-1.01) | 1.67 |
| H1g | 0.02 (0.02/0.01) | 1.03 (0.76-1.39) | 0.05 |
| H1h | 0.04 (0.04/0.04) | 1.04 (0.86-1.25) | 0.12 |
| H1i | 0.04 (0.04/0.04) | 1.04 (0.86-1.28) | 0.17 |
| H1j | 0.04 (0.04/0.05) | 0.85 (0.70-1.03) | 1.48 |
| H1l | 0.03 (0.04/0.03) | 1.17 (0.95-1.43) | 0.91 |
| H1m | 0.03 (0.02/0.03) | 0.82 (0.64-1.05) | 1.46 |
| H1o | 0.02 (0.02/0.02) | 0.98 (0.74-1.29) | 0.12 |
| H1p | 0.01 (0.01/0.01) | 1.45 (1.01-2.08) | 2.00 |
| H1r | 0.02 (0.03/0.02) | 1.31 (1.03-1.69) | 1.86 |
| H1s | 0.02 (0.01/0.02) | 0.84 (0.62-1.14) | 1.03 |
| H1u | 0.05 (0.05/0.06) | 0.94 (0.79-1.11) | 0.49 |
| H1x | 0.01 (0.02/0.01) | 1.21 (0.88-1.65) | 0.75 |
| H1y | 0.01 (0.02/0.01) | 1.16 (0.85-1.58) | 0.66 |
| N/A | 0.01 (0.01/0.01) | 1.10 (0.80-1.52) | 0.27 |
|  |  |  |  |
| **OR** = Odds ratio; **CI** = Confidence interval | |  |  |
