# Supplementary material for: 17q21.31 sub-haplotypes underlying H1-associated risk for Parkinson’s disease are associated with LRRC37A/2 expression in astrocytes
Source: Mol Neurodegener. 2022 Jul 15;17:48. doi: 10.1186/s13024-022-00551-x (PMC9284779; doi:10.1186/s13024-022-00551-x)
Supplement: Supplementary file 11 — Additional file 11. Supplementary table 4 [file 13024_2022_551_MOESM11_ESM.docx]

| **Block** | **Sub-haplotype ID** | **Sub-haplotype** | **WGS Frequency** |
| --- | --- | --- | --- |
| H1.1 | H1.1a | ACTCT | 0.3 |
|  | H1.1b | ACTTG | 0.23 |
|  | H1.1c | GCCTG | 0.19 |
|  | H1.1d | ATCTG | 0.23 |
|  | H1.1e | ACCTG | 0.05 |
|  |  |  |  |
| H1.2 | H1.2a | TTTCGATG | 0.39 |
|  | H1.2b | TCTCGATG | 0.18 |
|  | H1.2c | TTAAAATA | 0.19 |
|  | H1.2d | TTAAGATG | 0.08 |
|  | H1.2e | CTTCGATG | 0.08 |
|  | H1.2f | TTTCGGTG | 0.05 |
|  | H1.2g | TTAAAATG | 0.01 |
|  | H1.2h | TTTCGACG | 0.02 |
|  |  |  |  |
| H1.3 | H1.3a/H1.3c* | GACTGAGAT/GATTGAGAT | 0.45 |
|  | H1.3b | CATTAGGGC | 0.18 |
|  | H1.3d | CTTTGGTGC | 0.19 |
|  | H1.3e | CATTGGGGC | 0.04 |
|  | H1.3f | CATTGGGGT | 0.08 |
|  | H1.3g | GATCGAGAT | 0.06 |

**Table S4. Sub-haplotype frequencies in WGS data from AMP-PD**

*SNP rs72836318 (third variant in the block) did not pass QC and

was not present in the analyzed dataset. It was therefore not possible

to distinguish frequencies between H1.3a and H1.3c in these data
