# Supplementary material for: 17q21.31 sub-haplotypes underlying H1-associated risk for Parkinson’s disease are associated with LRRC37A/2 expression in astrocytes
Source: Mol Neurodegener. 2022 Jul 15;17:48. doi: 10.1186/s13024-022-00551-x (PMC9284779; doi:10.1186/s13024-022-00551-x)
Supplement: Supplementary file 12 — Additional file 12. Supplementary table 5 [file 13024_2022_551_MOESM12_ESM.docx]

**Table S5. Sample summary for RNA-seq expression analyses, dPCR copy number variation analyses and qRTPCR.**

|  |  | **Brain Region/Tissue** | **N** | **#H1H1** | **#Homozygote block1 H1 sub-haplotypes** | **#Homozygote block2 H1 sub-haplotypes** | **#Homozygote block3 H1 sub-haplotypes** | | **#H2H2** |
| --- | --- | --- | --- | --- | --- | --- | --- | --- | --- |
| RNA-seq Analysis | AMP-AD ROSMAP | PFC | 450 | 289 | 64 | 61 | 56 | 19 | |
|  | AMP-AD MAYO | TCX | 276 | 185 | 49 | 45 | 38 | 7 | |
|  | CommonMind | PFC | 624 | 347 | 70 | 34 | 28 | 21 | |
|  | Total | - | 1350 | 821 | 183 | 140 | 122 | 47 | |
|  |  |  |  |  |  |  |  |  | |
| dPCR | MSMD | Blood | 66 | 49 | 37 | 30 | 32 | 17 | |
|  | ADRC | Blood | 33 | 21 | 16 | 9 | 13 | 12 | |
|  | Charney LB | Blood | 20 | 17 | 13 | 12 | 9 | 3 | |
|  | Charney PD | PFC | 41 | 34 | 18 | 18 | 15 | 4 | |
|  | Total | - | 160 | 121 | 84 | 69 | 69 | 36 | |
|  |  |  |  |  |  |  |  |  | |
| qRT-PCR | Charney PD | PFC | 41 | 34 | 18 | 18 | 15 | 4 | |

**PFC** = prefrontal cortex; **TCX** = temporal cortex; **AMP-AD** = Accelerating Medicines Partnership Alzheimer’s disease; **ROSMAP** = Religious Orders Dtudy and Memory and Aging Project; **MSMD** = Mount Sinai Movement Disorders; **ADRC** = Alzheimer’s Disease Research Center; **LB** = Living brain; **PD** = Parkinson’s disease
