# Supplementary material for: 17q21.31 sub-haplotypes underlying H1-associated risk for Parkinson’s disease are associated with LRRC37A/2 expression in astrocytes
Source: Mol Neurodegener. 2022 Jul 15;17:48. doi: 10.1186/s13024-022-00551-x (PMC9284779; doi:10.1186/s13024-022-00551-x)
Supplement: Supplementary file 13 — Additional file 13. Supplementary table 6 [file 13024_2022_551_MOESM13_ESM.docx]

**Table S6. Quality control summary for Stage 1 and Stage 2 PD data analysis**

|  | **Stage 1** | | | | | **Stage 2** | | |
| --- | --- | --- | --- | --- | --- | --- | --- | --- |
|  | NIH | NL | GER | FIN | Total | McGILL | SPAIN | Total |
| Initial sample size | 4005 | 2796 | 1686 | 883 | 9370 | 1847 | 3444 | 5291 |
| Missingness | 37 | 3 | 10 | 21 | 71 | 5 | 0 | 5 |
| Relatedness | 6 | 68 | 3 | 2 | 79 | 0 | 0 | 0 |
| Ancestry | 56 | 0 | 0 | 0 | 56 | 0 | 0 | 0 |
| Missing phenotype | 0 | 0 | 0 | 0 | 0 | 357 | 0 | 357 |
| Excluded (n) | 99 | 71 | 13 | 23 | 206 | 362 | 0 | 362 |
| Excluded (%) | 2.47 | 2.54 | 0.77 | 2.60 | 2.20 | 19.60 | 0.00 | 19.60 |
|  |  |  |  |  |  |  |  |  |
| Final sample size | **3906** | **2725** | **1673** | **860** | **9164** | **1485** | **3444** | **4929** |
| Cases | 905 | 767 | 740 | 368 | 2780 | 582 | 2117 | 2699 |
| Controls | 3001 | 1958 | 933 | 492 | 6384 | 903 | 1327 | 2230 |
