# Supplementary material for: 17q21.31 sub-haplotypes underlying H1-associated risk for Parkinson’s disease are associated with LRRC37A/2 expression in astrocytes
Source: Mol Neurodegener. 2022 Jul 15;17:48. doi: 10.1186/s13024-022-00551-x (PMC9284779; doi:10.1186/s13024-022-00551-x)
Supplement: Supplementary file 14 — Additional file 14. Supplementary table 7 [file 13024_2022_551_MOESM14_ESM.docx]

**Table S7. dPCR probe design**

| **Probe ID** | **Source** | **Reference/Catalog ID** | **Label** | **Forward** | **Reverse** | **Probe** |
| --- | --- | --- | --- | --- | --- | --- |
| Alpha | ThermoFisher | Hs03955205_cn | FAM | - | - | - |
| Beta | ThermoFisher | Hs03971091_cn | FAM | - | - | - |
| Gamma | Custom | Boettger et al 2012 | FAM | GTTGTTGACCATGGCTTCCT | GTGAGAAGACGGCCTTTGAG | CACATGTGTTCTGGAATGCC |
| *LRRC37A* | Custom | - | FAM | TGTGTGTGTGTGTGTGTTTGTG | CTGCTCTGCTTTCATTCAAACCTTT | TTTCCTTTTTGTGTCCATCTCTCTCC |
| *MAPT* | ThermoFisher | Hs07226271_cn | FAM | - | - | - |
| RNase P | ThermoFisher | 4403326 | VIC | - | - | - |
