# Supplementary material for: 17q21.31 sub-haplotypes underlying H1-associated risk for Parkinson’s disease are associated with LRRC37A/2 expression in astrocytes
Source: Mol Neurodegener. 2022 Jul 15;17:48. doi: 10.1186/s13024-022-00551-x (PMC9284779; doi:10.1186/s13024-022-00551-x)
Supplement: Supplementary file 15 — Additional file 15. Supplementary table 8 [file 13024_2022_551_MOESM15_ESM.docx]

**Table S8. Summary of iPSC line sources and 17q21.31 haplotypes.**

| **Line ID** | **Source** | **Sex** | **Haplotype** |
| --- | --- | --- | --- |
| F11349 | ADRC | Male | H2H2 |
| F0510.2Δ2H1 | ADRC | Male | H1H1 |
| FA12455 | ADRC | Female | H2H2 |
| F13505 | ADRC | Female | H1H1 |
| 3182-3 | NIHCZ | Female | H2H2 |
| F11421Δ2A07 | ADRC | Female | H1H1 |
| UILK | NYSCF | Male | H2H2 |
| EIPL | NYSCF | Female | H2H2 |
| GP1.1 | NSWBB | Female | H1H1 |

**ADRC** = Knight Alzheimer’s Disease Research Center at

Washington University; **NIHCZ** = NIH Childhood-onset

Schizophrenia study. **NYSCF** = New York Stem Cell Foundation.

**NSWBB** = New South Wales Brain Bank
